# *In-silico* drug trials for precision medicine in atrial fibrillation: From ionic mechanisms to electrocardiogram-based predictions in structurally-healthy human atria

**DOI:** 10.3389/fphys.2022.966046

**Published:** 2022-09-15

**Authors:** Albert Dasí, Aditi Roy, Rafael Sachetto, Julia Camps, Alfonso Bueno-Orovio, Blanca Rodriguez

**Affiliations:** ^1^ Department of Computer Science, University of Oxford, Oxford, United Kingdom; ^2^ Departamento de Ciência da Computação, Universidade Federal De São João Del-Rei, São João del Rei, Brazil

**Keywords:** atrial fibrillation, *in-silico*, drug trials, cardioversion, electrocardiogram

## Abstract

Atrial fibrillation (AF) inducibility, sustainability and response to pharmacological treatment of individual patients are expected to be determined by their ionic current properties, especially in structurally-healthy atria. Mechanisms underlying AF and optimal cardioversion are however still unclear. In this study, in-silico drug trials were conducted using a population of human structurally-healthy atria models to 1) identify key ionic current properties determining AF inducibility, maintenance and pharmacological cardioversion, and 2) compare the prognostic value for predicting individual AF cardioversion of ionic current properties and electrocardiogram (ECG) metrics. In the population of structurally-healthy atria, 477 AF episodes were induced in ionic current profiles with both steep action potential duration (APD) restitution (eliciting APD alternans), and high excitability (enabling propagation at fast rates that transformed alternans into discordant). High excitability also favored 211 sustained AF episodes, so its decrease, through prolonged refractoriness, explained pharmacological cardioversion. In-silico trials over 200 AF episodes, 100 ionic profiles and 10 antiarrhythmic compounds were consistent with previous clinical trials, and identified optimal treatments for individual electrophysiological properties of the atria. Algorithms trained on 211 simulated AF episodes exhibited >70% accuracy in predictions of cardioversion for individual treatments using either ionic current profiles or ECG metrics. In structurally-healthy atria, AF inducibility and sustainability are enabled by discordant alternans, under high excitability and steep restitution conditions. Successful pharmacological cardioversion is predicted with 70% accuracy from either ionic or ECG properties, and it is optimal for treatments maximizing refractoriness (thus reducing excitability) for the given ionic current profile of the atria.

## 1 Introduction

In-silico trials with human modeling and simulation represent an effective means for drug safety and efficacy assessment (Passini et al., 2017; Musuamba et al., 2021; Margara et al., 2022). Besides yielding accurate predictions of pro-arrhythmic toxicity, superior to that obtained through animal studies (Passini et al., 2017), they provide mechanistic explanations to potential adverse events. Accordingly, in-silico trials constitute a central paradigm for drug safety (Passini et al., 2021), with unexploited capabilities for drug efficacy evaluation at higher dimensional levels (i.e., whole-organ scale) (Margara et al., 2022). The latter could support the introduction of human-based multiscale modeling and simulation into precision medicine, as shown by Roney et al., 2022 for the prediction of atrial fibrillation (AF) recurrence after catheter ablation. Similarly, in-silico trials offer the possibility of identifying key modulators of successful pharmacological treatment, that could guide tailored AF therapies.

Recent studies of AF pathophysiology have highlighted the low incidence (i.e., 3% of cases) of AF in the absence of heart disease (Wyse et al., 2014). Newly identified forms of heart disease, however, coincide with the absence of atrial structural abnormalities. Channelopathies (Wyse et al., 2014), genetic variants (Ragab et al., 2020) metabolic disorders, imbalanced autonomic nervous system (Hanna et al., 2021), endocrine control (Aguilar et al., 2021) or the inflammatory response (Scott et al., 2021), might increase AF susceptibility by transiently altering the electrical substrate (Heijman, Linz and Schotten, 2021). These dynamic substrates, as opposed to static structural inhomogeneities, might underlie the high variability observed across AF paroxysms. While patients presenting the above-mentioned disorders cannot be categorized as healthy, they might have structurally-healthy atria, in which AF inducibility has proven to be common, especially in males (Kumar et al., 2012).

*In-vivo* studies conducted in human subjects (Kim et al., 2002; Narayan et al., 2008; Krummen et al., 2012) observed that dynamic substrates favoring AF initiation resulted from steep action potential duration (APD) restitution (APDR). Steep APDR favored APD alternans and dynamic heterogeneities in repolarization when the atria were paced at rapid rates. Simulation studies (Gong et al., 2007) showed that discordant APD alternans (i.e., APD alternating in a beat-to-beat short-long pattern, with opposing phase in neighboring regions) could derive from steep APDR and preceded AF initiation. While discordant alternans was only observed for elevated L-type Ca^2+^ current, the authors did not analyze further alterations. Thus, other ionic current properties, mechanisms or channel dysregulation, resulting from genetic polymorphisms (Ragab et al., 2020), imbalanced regulatory systems (Heijman, Linz and Schotten, 2021) or natural inter-subject variability in ionic densities (Muszkiewicz et al., 2018), may also engage with steep electrical restitution and facilitate AF induction in patients with structurally-healthy atria. The mechanisms are however unknown.

Early detection of AF in patients with structurally-healthy atria is crucial, given their higher eligibility for antiarrhythmic drug treatment (Andrade et al., 2020; Hindricks et al., 2021). Indeed, absence of structural heart disease and AF duration <24 h are independent predictors of successful pharmacological cardioversion to normal sinus rhythm (Boriani et al., 2004). Restoring sinus rhythm limits the atrial structural remodeling (Andrade et al., 2020) and reduces the risk of stroke and cardiovascular death compared to rate control strategies (Kirchhof et al., 2020). Nevertheless, the success rate of pharmacological treatment is currently suboptimal (Heijman, Linz and Schotten, 2021), and little is known about how inter-subject variability in channel densities (Muszkiewicz et al., 2018) determines the response of individual AF patients to antiarrhythmic drugs (Capucci et al., 2018).

This lack of knowledge might be attributed to the unfeasible, non-ethical and risky nature of studying the effect of inter-subject variability in human electrophysiology on drug efficacy. The latter would require the patient to undergo multiple treatments and invasive screening procedures. Modeling and simulation studies, on the other hand, can provide strong evidence under high spatio-temporal resolution and perfect control over the parameters of interest, avoiding all three barriers and overcoming experimental limitations (Heijman et al., 2021).

Therefore, the aim of this study is to exploit human-based multiscale modeling and simulation to 1) identify the ionic profiles that determine electrical restitution and excitability properties, and favor AF in structurally-healthy atria, 2) understand their relationship with pharmacological cardioversion, and 3) quantify the prognostic significance for successful pharmacological cardioversion of ionic current densities versus electrocardiogram (ECG) metrics. To address these goals, simulated AF episodes were induced in a population of human-based whole-atria models with varying ionic densities and steep electrical restitution properties, as observed *in-vivo*. Pharmacological cardioversion was subsequently attempted by simulating the effects of the antiarrhythmic drugs used clinically for restoring sinus rhythm. Simulation results were consistently evaluated against experimental and clinical data. Ultimately, predictive algorithms for AF cardioversion, in the form of clinical decision support systems, were built using the information obtained from ECG metrics and ionic current profiles.

## 2 Methods

### 2.1 Population of human atrial cardiomyocyte models

A population of human atrial cardiomyocyte models was generated as described in Muszkiewicz et al., 2018. The CRN model (Courtemanche, Ramirez and Nattel, 1998) was considered as baseline, since it reproduces the long plateau observed in paroxysmal AF patients with steep APDR (Narayan et al., 2008; Krummen et al., 2012) and it has been extensively used for developing and testing antiarrhythmic drug modeling (Loewe et al., 2014, 2015; Sutanto et al., 2019). Effects of drug modeling on the CRN model, moreover, were compared to the effects on the Grandi-Bers (Grandi et al., 2011) and Maleckar-Trayanova (Maleckar et al., 2009) models to assess model independence of key results.

Key conductances and permeabilities were sampled up to ±70% of their control ranges using Latin Hypercube sampling, including the ultrarapid, rapid and slow delayed-rectifier K^+^ current density (G_Kur_, G_Kr_ and G_Ks_), transient outward K^+^ current density (G_to_), inward rectifier K^+^ current density (G_K1_), L-type Ca^2+^ current density (G_CaL_), fast Na^+^ current density (G_Na_), Na^+^/K^+^ pump (G_NaK_), Ca^2+/^Na^+^ exchanger (G_NCX_) and the sarcoplasmic reticulum Ca^2+^ release (G_rel_), leak (G_leak_) and uptake (G_up_) currents. Experimental calibration was subsequently applied using action potential characteristics obtained from human cells of patients in sinus rhythm (dataset and further explanation available in Sánchez et al., 2014; Table 1). No AF remodeling was applied to the calibrated population of atrial cardiomyocyte models.

**TABLE 1:** Prediction accuracy (mean and confidence interval). Most relevant features selected by the elastic-net logistic regression. Abbreviations: **DF**: Dominant frequency; **SaE**: Sample entropy. **RHE**: Relative harmonic energy.

Drug	Data	Best predictive biomarkers	Accuracy (%)
Dronedarone	ECG	DF (Leads V2–V6), SaE (Leads V2, V6)	0.72 [0.70, 0.74]
	Ionic properties	GK1, GKur, GNaK, GNa, GKr, GCaL	0.76 [0.74, 0.78]
	ECG + Ionic	CV, DF (Leads V2, V4)	0.77 [0.75, 0.79]
Flecainide	ECG	SaE (Lead V1), RHE (Lead III), SaE (Lead III)	0.69 [0.67, 0.71]
	Ionic properties	GNaK, GK1, GKs, Gto	0.71 [0.69, 0.73]
	ECG + Ionic	Gto, GK1, GNaK, GKs, SaE (Lead V1)	0.72 [0.70, 0.74]
Vernakalant	ECG	RHE (Lead V4, V5), SaE (V6)	0.68 [0.66, 0.70]
	Ionic properties	Gto, GKur, GNaK	0.70 [0.68, 0.72]
	ECG + Ionic	Gto, GKur, SaE (Lead V6), RHE (Lead V4, V5)	0.70 [0.68, 0.72]

The APDR curves of the experimentally-calibrated population were computed by both standard S1S2 and dynamic restitution protocols. The standard S1S2 protocol consisted of a train of stimuli applied at a fixed S1 cycle length (CL), followed by a single extrastimulus with coupling interval S2. For the dynamic restitution protocol, the train was applied until steady-state at constant S1, for decreasing CLs. Conduction velocity (CV) restitution (CVR) was measured for models yielding APDR slope greater than unity for both restitution protocols (Koller, Riccio and Gilmour, 1998; Watanabe et al., 2001), and an APDR slope greater than unity over a diastolic interval range (Qu, Weiss and Garfinkel, 1999) of at least 30 milliseconds (ms). A detailed explanation of the APDR and CVR calculation can be found in the Supplementary Material.

### 2.2 Populations of whole-atria models with homogeneous vs. heterogeneous ionic current properties

Ninety-seven atrial cardiomyocyte models presenting steep APDR and CVR were used to populate two human-based whole-atria models, one with homogeneous and one with heterogeneous ionic current properties (Supplementary Figure S1). The population of 97 homogeneous whole-atria models was constructed by assigning one atrial cardiomyocyte model to all regions of the atria (to isolate the contribution of ionic densities to AF). The population of 97 heterogeneous models was similarly constructed with each atrial cardiomyocyte model, but in this case, the ionic profile was modified in specific atrial regions, as previously detailed (Seemann et al., 2006; Tobón et al., 2013; Sánchez et al., 2017). Specifically, the single-cell properties of the cardiomyocyte model were assigned to the left atrial tissue, so that the restitution properties interfered with burst pacing at the pulmonary veins (see below), and scaled in the right atrium, crista terminalis, pectinate muscles, left atrial appendage and atrio-ventricular rings, as detailed in Supplementary Table S1.

Regional heterogeneities in CV and anisotropy ratio were included in both populations of whole-atria models as in (Tobón et al., 2013; Sánchez et al., 2017) (Detailed in Supplementary Table S1). Simulations of electrical propagation were conducted using the monodomain equation with the MonoAlg3D software (Sachetto Oliveira et al., 2018; available at https://github.com/rsachetto/MonoAlg3D_C).

### 2.3 Atrial fibrillation inducibility

The protocol for simulated AF induction mimicked the spontaneous ectopic beats reported in human patients with frequent episodes of AF (Haïssaguerre et al., 1999). As such, a burst of five periodic stimuli was applied at the left pulmonary veins, the most common foci origin site. To account for repetitive discharges with irregular CL, different CLs, ranging from 170 to 260 ms (Haïssaguerre et al., 1999; mean CL of focal discharges 175 ± 30 msec) with 10 ms increment, were tested for the burst. AF episodes longer than 3 s were considered sustained.

### 2.4 Atrial fibrillation pharmacological management: Drug modeling

Sustained AF episodes were subjected to 10 simulated pharmacological treatments: five drugs currently approved for restoring sinus rhythm (i.e., flecainide, propafenone, vernakalant, amiodarone, and ibutilide; Hindricks et al., 2021), dronedarone (an analogue of amiodarone), ranolazine (as a promising compound for sinus rhythm maintenance; Capucci et al., 2018) and digoxin (a rate control agent previously used for rhythm control; The Digitalis in Acute Atrial Fibrillation (DAAF) Trial Group, 1997). Drug action was simulated as simple pore-block models according to previously reported effective plasma drug concentration, and their 50% inhibitory concentration and hill coefficient profiles (Supplementary Table S2, Supplementary Figure S2). For computational feasibility, considering the large number and complexity of the simulations and the deterministic nature of the models, drug administration was modelled 3 s from AF induction (Sánchez et al., 2017), and the episode was recorded for another 4 s. AF was considered successfully cardioverted by the drug if the atria were free of arrhythmic activity before the completion of the 7 s (Matene and Jacquemet, 2012). Drug efficacy was defined as the number of AF episodes cardioverted by a given drug over the total number of AF episodes.

Moreover, to evaluate whether pharmacological AF prevention and cardioversion followed the same restitution mechanisms, the AF-induction protocol was repeated in sinus rhythm after drug application. For this, only the two drugs that steepened/flattened the APDR curve the most were considered.

### 2.5 Simulated electrocardiogram

Simulated 8-lead (leads I, II, V1-V6) ECGs were computed for sustained fibrillation as in Gima and Rudy, 2002. The ECG was recorded during the 3-s AF episode prior to drug application. The resulting signals were standardized and processed using pass-band filtering with cut-off frequency of [0.5–50] Hz (Alcaraz et al., 2011). Five biomarkers, namely, dominant frequency, organization index, Shannon’s spectral entropy, sample entropy and relative harmonic energy, were extracted from each lead as in (Alcaraz et al., 2011; Zeemering et al., 2018). These biomarkers provided a characterization of AF complexity in the frequency and temporal domain.

### 2.6 Clinical decision support systems

An algorithm for predicting the likelihood of pharmacological cardioversion (equivalent to a clinical decision support system) was built using ECG metrics, ionic current profiles and cellular electrophysiological properties as input. For this, a dataset was constructed for each antiarrhythmic drug, combining the information of the AF episodes and the outcome of drug application. Every AF episode was characterized by M = 55 features, including 15 derived from the electrophysiological properties of the atria (i.e., baseline CV, APD_90_, resting membrane potential (RMP) and 12 ionic current densities) and 40 from the ECG analysis (i.e., 5 biomarkers from each lead, 8-lead ECG). Each episode was labelled either as 1, if AF was successfully cardioverted, or 0, otherwise.

The whole dataset was partitioned in sub-datasets to determine the predictive power of each set of biomarkers. Clinical decision support systems were built with each sub-dataset by elastic-net logistic regression (Zeemering et al., 2018), using the Glmnet toolbox (available at http://hastie.su.domains/glmnet_matlab/). Parameter selection was done by stepwise logistic regression with alpha equal 0.5 and 100 values for lambda. The performance of the predictive models was validated through 5-fold cross-validation, repeated 20 times, randomly allocating 70–30% of data for training and testing, respectively. Further details of the algorithms and the different sub-datasets can be found in the Supplementary Material.

## 3 Results

### 3.1 Evaluation of human atrial cardiomyocyte action potential models shows that steep restitution is associated with low triangulation and high cellular excitability

**FIGURE 1:**
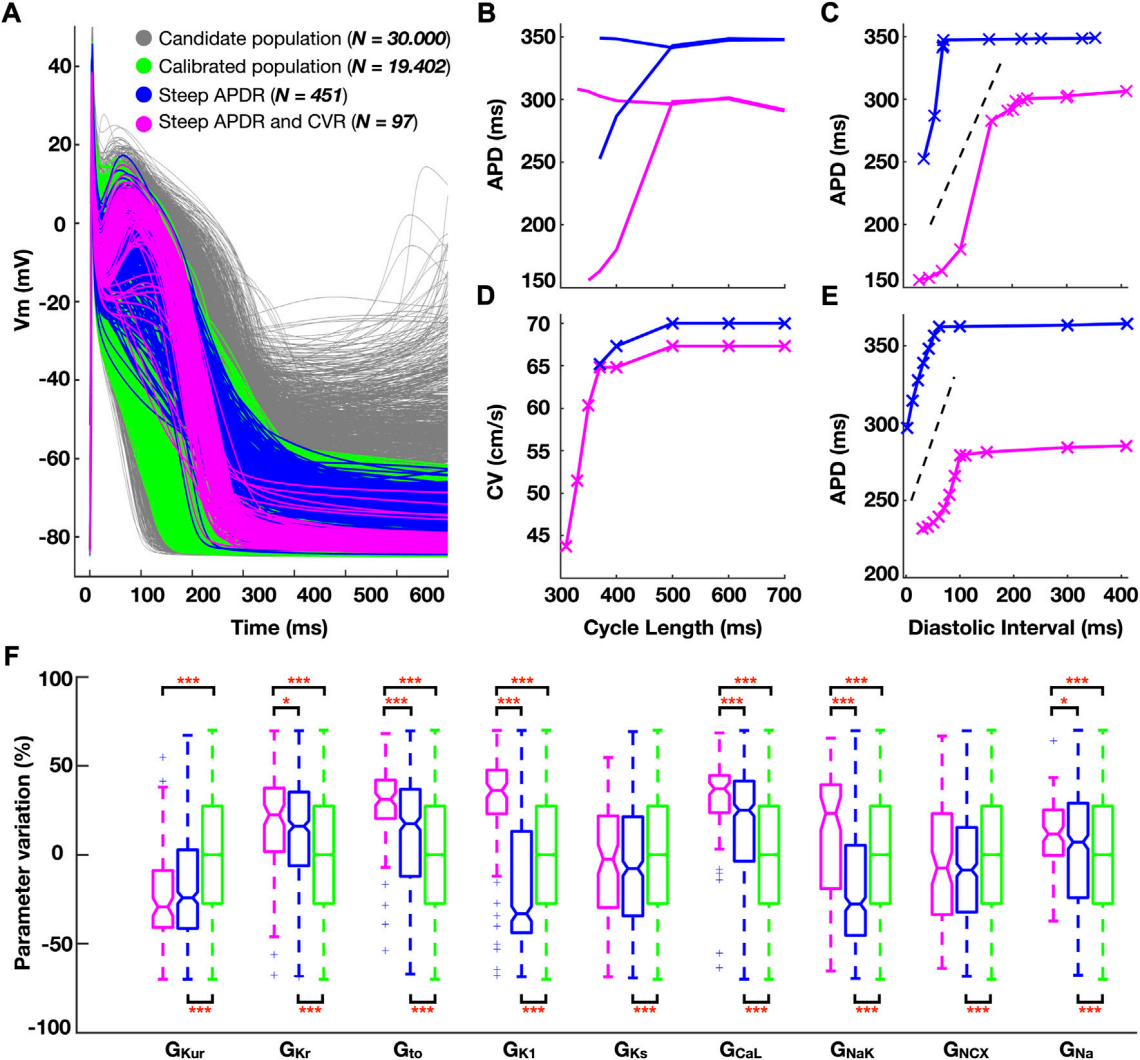
**(A)** Simulated action potential traces for human atrial cardiomyocyte models in the population before (grey) and after (green) experimental calibration. **(B–E)** Comparison of the electrical restitution between a representative cardiomyocyte model characterized by steep action potential duration restitution (APDR) alone (blue) and additional steep conduction velocity restitution (CVR, pink). **(B)** Dynamic APDR plotted against the cycle length, showing transient alternation, and **(C)** against the diastolic interval. **(D)** CVR. **(E)** Standard S1S2 APDR. **(F)** Ionic density distribution (color-code as per panel **(A)**. Data analyzed using Wilcoxon test. *p < 0.05, **p < 0.01, ***p < 0.001.

A calibrated subpopulation of human atrial cardiomyocyte models, matching experimental action potential characteristics, was selected from the initial candidate population. Four hundred fifty-one cardiomyocyte models in the calibrated subpopulation showed steep APDR for both restitution protocols, and 97 additionally presented steep CVR. Figure 1 illustrates action potential morphologies (Figure 1A), representative APDR and CVR curves of steep restitution properties (Figures 1B–E) and ionic density distribution (Figure 1F) of the subpopulations of atrial cardiomyocyte models considered.

Steep APDR curves occurred for longer plateau phases (triangulation (APD_90_-APD_50_) 72.6 ± 18.1 vs 126.9 ± 61.3 ms, steep vs flat APDR curves). Models additionally yielding steep CVR curves had a more negative resting membrane potential (RMP: 82.4 ± 1.7 vs. -74.5 ± 3.3 mV; steep APDR and CVR vs steep APDR alone) (Figure 1A, pink versus blue traces). The hyperpolarization of the RMP allowed propagation for shorter CLs (307 ± 15 vs 538 ± 152 ms; steep APDR and CVR vs steep APDR alone), increasing the magnitude of APD alternans (Figure 1B) and favoring a greater modulation of CV (Figure 1D).

Steep APDR alone was found in models with significantly high G_CaL_ and low G_Kur_ compared to flat restitution (Figure 1F, blue vs green boxes). Steep APDR and CVR curves (Figure 1F, pink boxes) were associated with even greater G_CaL_, elevated G_Na_, and high density of repolarization currents, especially G_NaK_, G_K1_, G_Kr_ and G_to_. The 97 atrial cardiomyocyte models exhibiting steep APDR and CVR were used to develop the populations of human whole-atria models (Supplementary Figure S1).

### 3.2 Atrial fibrillation is facilitated by discordant APD alternans enabled by propagation at fast rates due to high excitability, for high L-type Ca^2+^ and inward rectifier K^+^ currents

**FIGURE 2:**
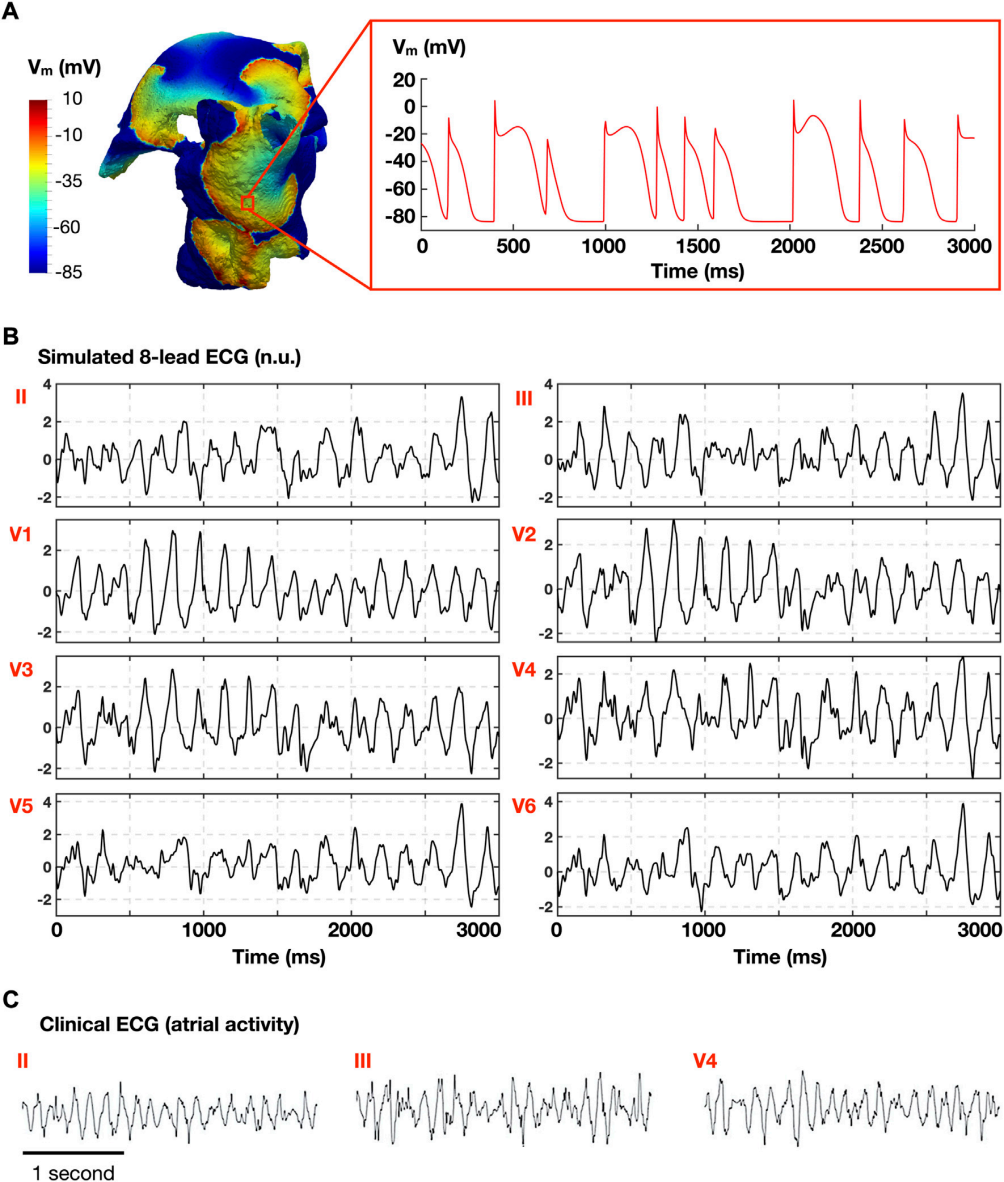
Representative atrial fibrillation (AF) episode. **(A)** Simulated AF in the whole atria model (left), and time course of the transmembrane voltage (V_m_) in 1 cell (right). **(B)** Simulated atrial electrocardiogram (normalized ECG; no units). **(C)** Clinical ECG obtained in AF patient (adapted from Lankveld et al., 2016 under permission).

212 and 265 AF episodes were induced in 75 out of the 97 homogeneous and heterogeneous whole-atria models, respectively, following burst stimulation at 10 different CLs. Whole-atria models with 22 ionic profiles failed to induce AF for both homogeneous and heterogeneous ionic current properties. Figure 2 illustrates a representative AF episode, showing multiple rotors in the whole-atria model (Figure 2A, left), the transmembrane voltage from 1 cell (Figure 2A, right), and similarities between the simulated ECG (atrial activity, Figure 2B) and the clinical ECG (atrial activity) obtained from a human AF patient (Figure 2C). More AF episodes were induced with the heterogeneous ionic substrate, but the 75 ionic profiles of the models ensuring AF inducibility were the same in both populations.

**FIGURE 3:**
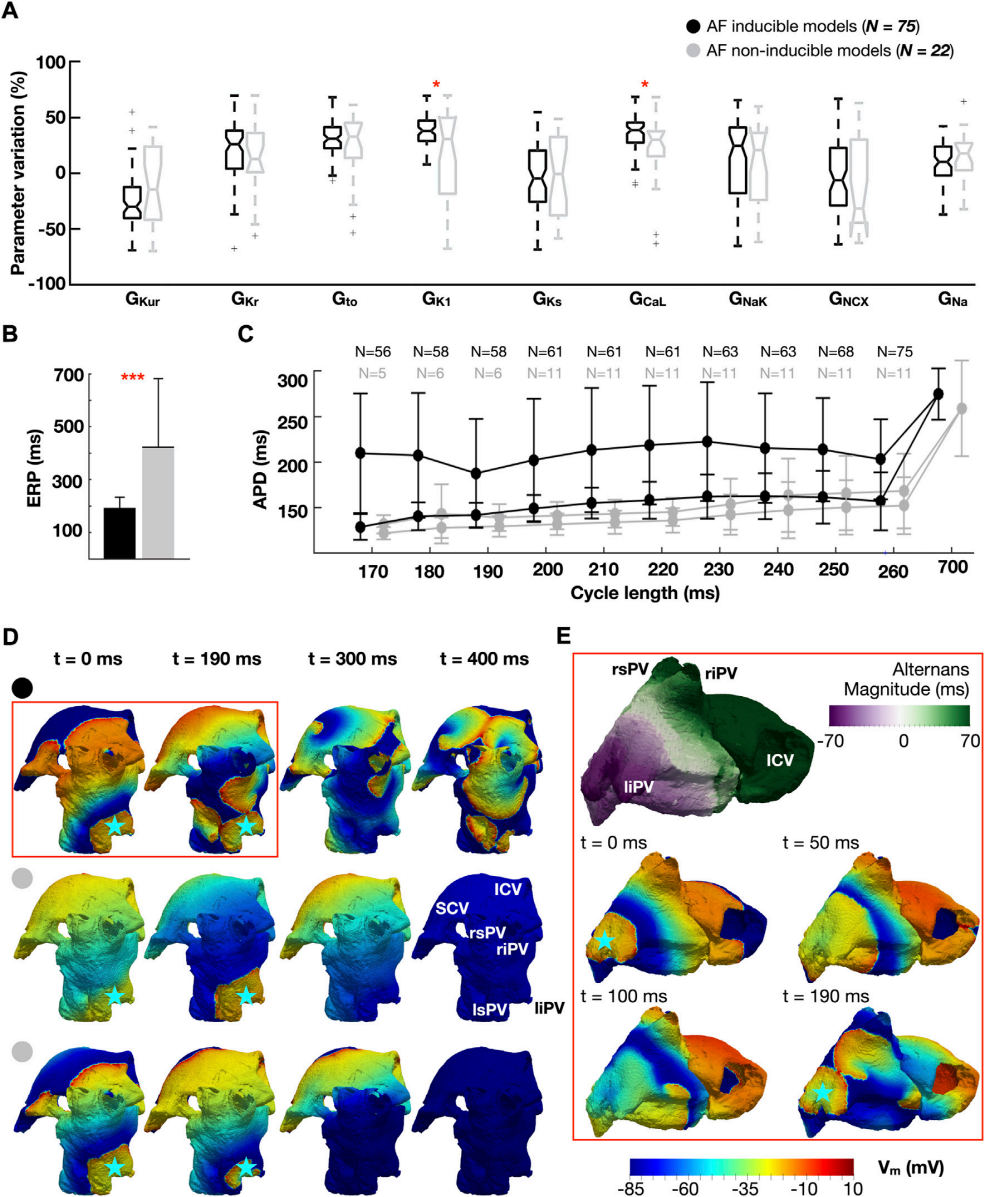
Ionic properties of AF-inducible (black) and non-inducible models (grey): **(A)** Ionic density distributions. **(B)** Effective refractory period (ERP). **(C)** Dynamic action potential duration (APD) restitution curves plotted against the cycle length used for burst pacing (170–260 ms). Two consecutive beats are considered (i.e., long and short APD), showing magnitude of APD alternans. Number (N) of models able to propagate all stimuli. **(D)** Comparison of propagation dynamics between an AF-inducible model (top), and two non-inducible models: one showing a 1:2 conduction pattern (middle row), the other propagating all stimuli without AF induction (bottom row). Burst stimulus applied at a cycle length of 190 ms. The blue star represents the location of the burst. **(E)** Discordant alternans favoring conduction block and re-entry. The snapshots in **(E)** are an expansion of the red box in **(D)**. Abbreviations. S-ICV: Superior and inferior cava vein; ri-rs-li-ls-PV: Right inferior, right superior, left inferior and left superior pulmonary veins. Data are expressed as medians and IQR and analyzed using Wilcoxon Test. *p < 0.05, **p < 0.01. ***p < 0.001.

Figure 3 illustrates the distribution of ionic densities (Figure 3A), effective refractory period (ERP) (Figure 3B), and APDR curves (Figure 3C) between the 75 atrial cardiomyocyte models favoring AF and the 22 non-inducible models. The dynamic restitution curves in Figure 3C are plotted against the CLs used for burst pacing (170–260 ms), for two consecutive beats to show magnitude of APD alternans (i.e., difference between long and short APD). Figures 3D,E compare the response of the whole-atria models to burst stimulation between AF inducible (Figure 3D, top and Figure 3E) and non-inducible models (Figure 3D, middle and bottom), to illustrate the dynamics underlying AF initiation. The analysis presented in Figure 3 considers the population with homogeneous ionic current properties.

Analysis of whole-atria simulations revealed AF inducibility for ionic profiles presenting high G_CaL_, which increased APD alternans, and G_K1_, which conferred high cellular excitability (Figures 3A–C). Indeed, all whole-atria models susceptible to AF exhibited propagation at CL = 260 ms, while this only occurred in 11 models (50%) free of arrhythmia (Figure 3C). Likewise, 56 (75%) AF inducible and 5 (23%) non-inducible whole-atria models enabled propagation at 170 ms (i.e., the shortest CL tested in the burst).

Figure 3D compares the response to burst stimulation between one whole-atria model susceptible to AF (top row) and two non-inducible models (one of them belonging to the 11 models that failed to propagate burst stimulation at CL = 260 ms (middle row), the other belonging to the 5 models that propagated at 170 ms without inducing AF (bottom row)). Whole-atria models unable to propagate all stimuli in the burst exhibited a 1:2 conduction pattern (i.e., propagation of one every two stimuli), which prevented engagement with the steep restitution properties, as illustrated in Figure 3D (middle): the stimulus at t = 190 ms propagates following propagation failure at t = 0 ms.

However, even when all burst stimuli propagated, some models were resistant to AF (Figure 3D, bottom). Analysis of their associated APDR curves revealed that AF-inducible models presented greater magnitude of APD alternans (67.3 ± 92.5 vs 8.9 ± 6.6 ms, AF-inducible vs non-inducible models) (Figure 3C). The latter developed into discordant alternans (i.e., APD alternating in a beat-to-beat short-long pattern, with opposing phase in neighboring regions) during the application of rapid burst stimulation, causing unidirectional block and re-entry at different sites of the left atrium (Figure 3E). An extensive explanation of discordant alternans formation after burst pacing is provided in the Supplementary Figure S3. Discordant alternans were not observed in whole-atria models free of AF.

Restoring the baseline density (i.e., ionic conductance) of the L-type Ca^2+^ current in AF-inducible models recapitulated the magnitude of APD alternans observed in non-inducible models (67.3 ± 92.5 vs 8.3 ± 6.0 ms, AF-inducible models with elevated vs control L-type Ca^2+^ current density). Similarly, alternans magnitude was reduced after decreasing the inward rectifier K^+^ current density to control conditions (67.3 ± 92.5 vs 18.2 ± 12.1; AF-inducible models with elevated vs control inward rectifier K^+^ current). The latter effect was due to a reduction in the number of models propagating at short CLs (explanation given in the Supplementary Figure S4).

### 3.3 Analysis of 477 AF episodes reveals atrial fibrillation sustainability supported by high excitability, with enhanced up-regulation of the inward rectifier K^+^ current and elevated Na^+^/K^+^ pump

AF sustainability was evaluated in the 75 whole-atria models ensuring AF inducibility considering homogeneous versus heterogeneous ionic current properties, to compare the role of the ionic profile and spatial gradients. The heterogeneous ionic substrate favored AF maintenance: 154 (58%) of the 265 AF episodes induced in heterogeneous models were sustained for over 3 s, versus 57 (27%) of 212 AF episodes induced in homogeneous models. In the former, the left atrial appendage and atrioventricular rings had a shorter APD_90_ than the bulk tissue, which favored rotor stabilization. Together with the APD heterogeneities within the right atrium (i.e., crista terminalis, pectinate muscles and bulk tissue) and between the right and left atrium, the heterogeneous ionic substrate presented inhomogeneous restitution properties that reinforced AF maintenance.

**FIGURE 4:**
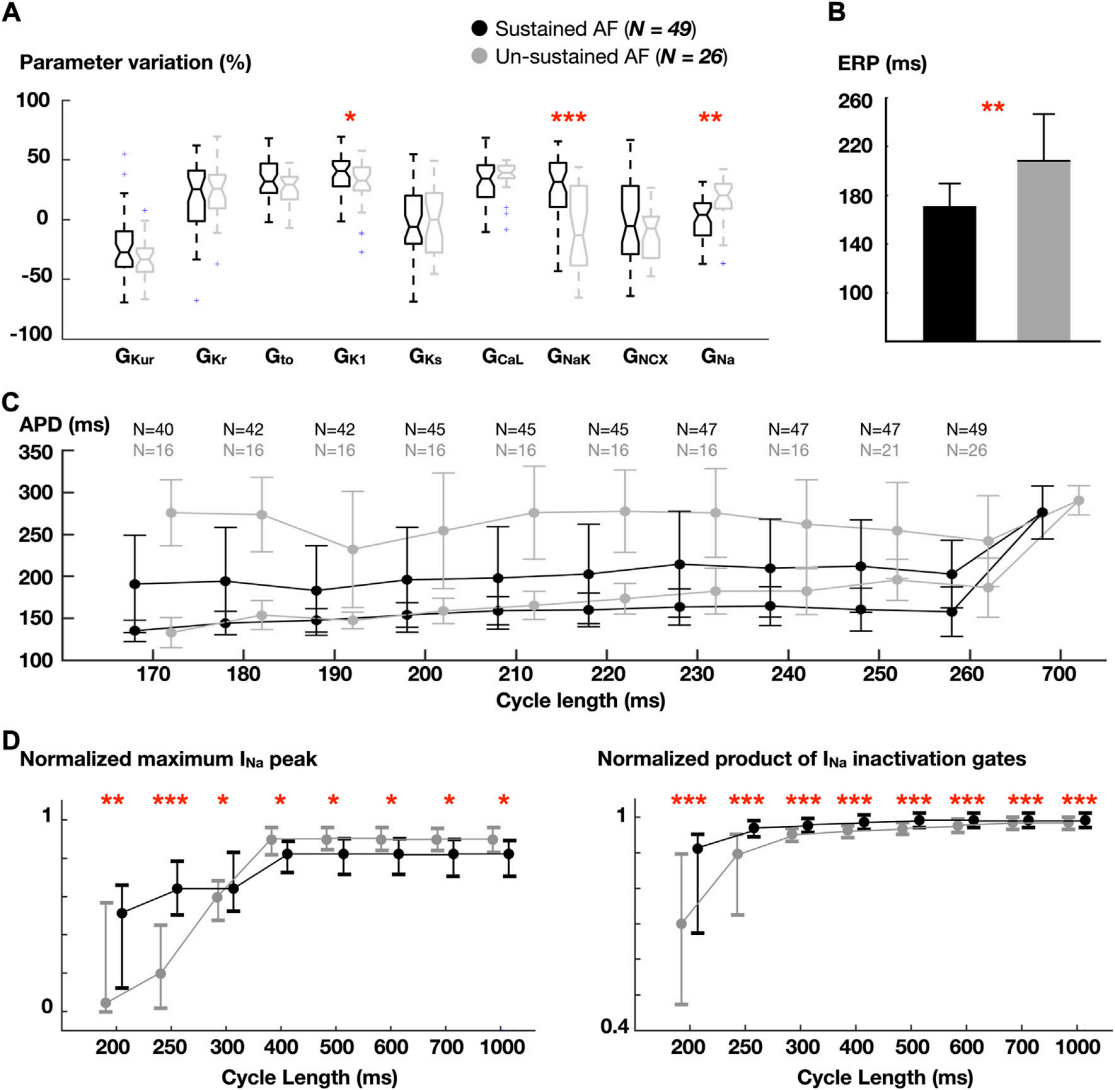
Comparison between atrial cardiomyocyte models favoring sustained vs un-sustained fibrillation in homogeneous whole-atria models. **(A)** Ionic density distribution. **(B)** Effective refractory period (ERP). **(C)** Dynamic action potential duration (APD) restitution (as in Figure 3C). Number (N) of models able to propagate all stimuli. **(D)** Normalized fast Na^+^ current (I_Na_) peak and normalized product of I_Na_ inactivation gates. Data are expressed as medians and IQR and analyzed using Wilcoxon Test. *p < 0.05, **p < 0.01, ***p < 0.001.

Overall, 211 (44%) AF episodes sustained in both populations of whole-atria models. In order to isolate the role of ionic profile rather than gradients in AF sustainability, the properties of the homogeneous models yielding sustained (>3s) and un-sustained fibrillation were analyzed. Thus, Figure 4 shows ionic density distribution (Figure 4A), ERP (Figure 4B), APDR curves (Figure 4C), and Na^+^ current dynamics (Figure 4D).

Higher G_NaK_ and G_K1_, and lower G_Na_ were associated with models sustaining AF. Analysis of Na^+^ inactivation gates revealed that, in spite of a lower G_Na_, Na^+^ availability was higher in the sustained AF group (Figure 4D, right). This was accentuated as the pacing rate increased: the maximum Na^+^ current peak, which was greater in the un-sustained AF group for lower rates, became higher in models yielding sustained AF when stimulated at short CLs (Figure 4D, left). This was due to the higher G_NaK_ and G_K1_, which hyperpolarized the RMP (-82.6 ± 0.7 vs. -81.5 ± 0.6 mV, sustained and un-sustained AF, respectively). Accordingly, these atrial cardiomyocyte models had higher cellular excitability (and shorter effective refractory period, Figure 4B).

At slow rates (CL = 700 ms), lower CVs were found in whole-atria models leading to sustained AF (60.3 ± 5.7 vs 62.5 ± 4.5 cm/s), as a result of lower G_Na_. At fast rates (CL = 260 ms), however, the opposite trend was observed (56.4 ± 9.8 vs 50.7 ± 5.7 cm/s; sustained vs unsustained AF). The magnitude of APD alternans was similar across groups.

### 3.4 In-silico drug trials in over 200 atrial fibrillation episodes consistently reproduce cardioversion efficacy observed in clinical trials and identify key properties determining treatment success

**FIGURE 5:**
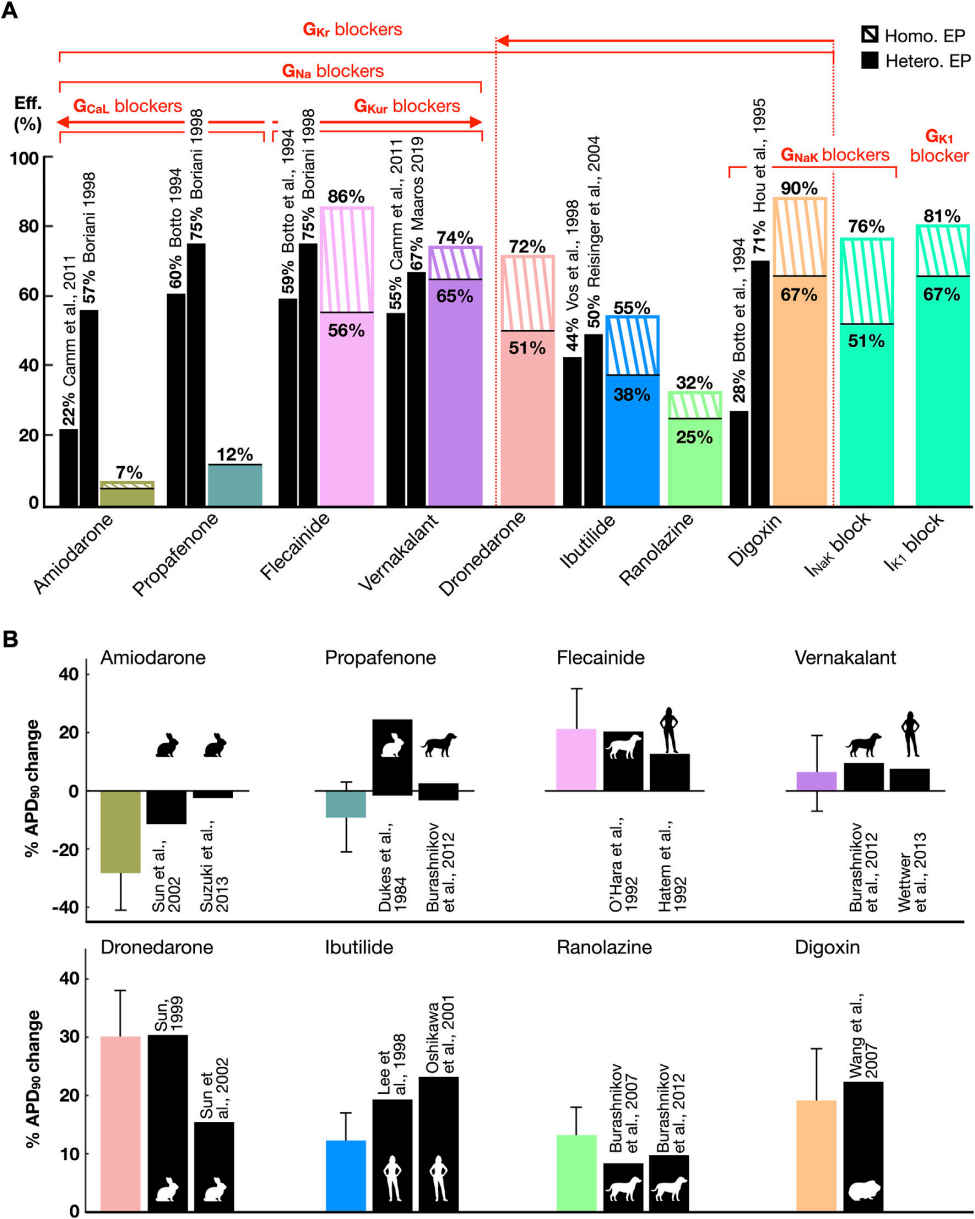
**(A)** Comparison between the cardioversion efficacy (Eff.) obtained in-silico (color bars) in whole-atria models with homogeneous (*Homo*.) and heterogeneous (Hete.) electrophysiology (EP) and human clinical trials (black bars). Drugs are grouped according to the ionic channels they target. A threshold of 25% block at therapeutic plasma concentration has been considered to denote ion current block. The arrows point towards greater ionic density block. **(B)** Percentage (%) of APD_90_ variation after drug modeling or administration with respect to control conditions, in-silico (color bars) and *in-vitro* (black bars, error bars *in-vitro* not available, see Supplementary Table S3). The animal silhouettes represent the species analyzed *in-vitro*.

Pharmacological cardioversion with 10 compounds was attempted in the 211 sustained AF episodes induced in both whole-atria populations (homogeneous, 58 episodes; heterogeneous, 153 episodes). Figure 5 displays the efficacy (Figure 5A) of each treatment obtained in-silico and reported in human clinical trials. In-silico antiarrhythmic drugs are displayed according to their preferential ionic channel target. Cardioversion efficacy was higher for homogeneous versus heterogeneous whole-atria models as illustrated by dashed and solid bars, respectively in Figure 5A (described below). Figure 5B compares the percentage (%) of APD_90_ variation obtained after drug action with respect to control conditions in-silico and *in-vitro*. Corresponding results were obtained for APD_90_ change between simulated drug action and experimental data (Figure 5B), with further validation, illustrating rate-dependence alterations in APD, CV, and maximum upstroke velocity, provided in Supplementary Table S3.

Moreover, a comparison of APD_90_ variation after drug action between the CRN, Grandi-Bers and Maleckar-Trayanova models can be found in the Supplementary Figure S5). The latter models showed a higher sensitivity to I_Kur_ inhibition than the CRN model, which presented a greater APD prolongation after I_Kr_ block. Thus, repeating the whole-atria simulations with the Grandi-Bers or Maleckar-Trayanova models is expected to produce even higher efficacy for I_Kur_ blockers than that already shown in Figure 5.

Higher cardioversion efficacy derived from class III (i.e., dronedarone and vernakalant) and class V (i.e., digoxin) antiarrhythmic drugs. Targeting I_Kr_, I_Kur_, I_NaK_ and I_K1_ yielded a greater prolongation of refractoriness than inward current block (Supplementary Figure S6). Antiarrhythmic drugs resulting in I_Kr_ inhibition (i.e, ranolazine, ibutilide, and dronedarone) showed an increase in cardioversion efficacy proportional to the extent of I_Kr_ block. However, higher success rates resulted from blocking multiple K^+^ channels, even for lower percentages of I_Kr_ inhibition. For instance, a 25% I_NaK_ and 40% I_Kr_ block (i.e., digoxin) was far more effective than 45% I_Kr_ (i.e., ibutilide, efficacy in-silico matching clinical trials) or 70% I_Kr_ block (i.e., dronedarone).

The high efficacy of digoxin, in agreement with some clinical trials, owed to I_NaK_ inhibition. Blocking I_NaK_ or I_K1_ alone raised the RMP and decreased I_Na_ availability. The subsequent prolongation of post-repolarization refractoriness was sufficient to terminate AF regardless of a subtle APD change (Supplementary Figure S6). Indeed, single I_K1_ block and digoxin were the pharmacological approaches that provided the highest cardioversion rates.

Combined I_Kr_ and I_Kur_ block (i.e., flecainide and vernakalant, whose cardioversion efficacies matched the efficacy reported in clinical trials) was also more effective than I_Kr_ inhibition alone, especially in the population with heterogeneous ionic current properties. In general, the heterogeneous substrate hampered pharmacological cardioversion, since it facilitated AF maintenance (Section 3.3) and antiarrhythmic drugs had an inhomogeneous effect on different atrial regions. In particular, the heterogeneous ionic substrate was characterized by a lower G_Kr_ in the right atrium compared to the left atrium (Supplementary Table S1), so that I_Kr_ inhibition lost cardioversion efficacy in this setting. Thus, the efficacy of flecainide and dronedarone, but not of vernakalant, dropped significantly in the heterogeneous ionic substrate.

Low cardioversion rates stemmed from targeting the inward currents, as a result of drug-induced APD reduction (Figures 5A,B). I_Na_ block was effective when complemented with I_Kur_ and I_Kr_ inhibition (i.e., flecainide and vernakalant). However, combined I_Na_ and I_CaL_ block (i.e., amiodarone and propafenone) shortened the APD, yielding the formation of focal re-entrant sources (Supplementary Figures S7, S8). The cardioversion efficacy decreased proportionally to I_CaL_ block (equivalently, resulting in larger APD abbreviation, Figures 5A,B), with amiodarone (i.e., 50% I_CaL_ and 30% I_Na_ block) showing the lowest cardioversion rate. The efficacy of amiodarone, and especially of propafenone, differed from the efficacy reported in clinical trials (see Discussion).

Nevertheless, amiodarone-induced APD shortening significantly flattened the APDR curved (Supplementary Figure S6). Investigations on cardio-protection instead of cardioversion (i.e., inducing the AF-initiation protocol after in-silico drug application) showed that amiodarone prevented 100% of recurrent AF episodes, with only atrial flutter (i.e., regular and periodic activation) remaining in 2% of cases. In contrast, dronedarone prolonged the APD, steepening the APDR. Therefore, while effective in preventing AF induced at very short CLs (i.e., 170–200 ms), 49% of AF episodes were still inducible in dronedarone-pretreated substrates.

### 3.5 Optimal pharmacological treatments for individual ionic profiles are those that maximize prolonged refractoriness thus minimizing excitability

**FIGURE 6:**
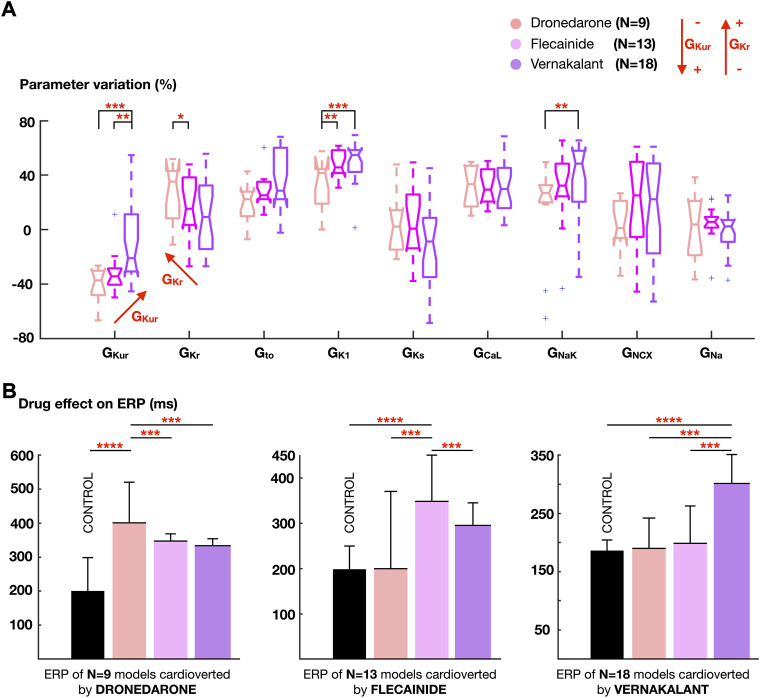
**(A)** Comparison of the ionic density distribution between atrial cardiomyocyte models showing favorable response only to dronedarone, flecainide or vernakalant. **(B)** Effect of the three antiarrhythmic drugs on the effective refractory period (ERP), stratifying the atrial cardiomyocyte models according to the pharmacological treatment that produced successful cardioversion. Colors on the bars as in **(A)**. Data are expressed as medians and IQR and analyzed using Wilcoxon Test. *p < 0.1, **p < 0.05, ***p < 0.01. ****p < 0.001.

Forty-nine AF episodes, sustained by 40 whole-atria models, were only successfully cardioverted by a single drug. Eleven episodes sustained in 9 models were only cardioverted by dronedarone, 14 episodes in 13 models by flecainide, and 24 episodes in 18 models by vernakalant. Figure 6 illustrates the ionic density distribution of the whole-atrial models that only responded favorably to dronedarone, flecainide, or vernakalant, and how the ERP of these models, grouped according to successful treatment, was modified after applying each drug.

The extent of ERP prolongation caused by each treatment in a particular model explained the ability for AF cardioversion. Thus, models showing a favorable response to dronedarone (*N* = 9) also presented a greater ERP prolongation after dronedarone application. Similarly, flecainide prolonged the ERP more than dronedarone or vernakalant in whole-atria models only cardioverted by flecainide (*N* = 13), and the same applied for vernakalant (Figure 6B).

Analysis of the ionic current profiles revealed that atrial cardiomyocyte models showing greater ERP prolongation and thus, successful cardioversion after vernakalant application, had high G_Kur_ and low G_Kr_. This agrees with vernakalant action, with the strongest I_Kur_ inhibitory profile within the tested antiarrhythmic drugs. By contrast, dronedarone cardioverted AF in models presenting the opposite situation: higher G_Kr_ and lower G_Kur_. Flecainide resulted more effective for an intermediate scenario, since it blocked G_Kur_ and G_Kr_, but to a lesser extent than vernakalant and dronedarone, respectively (Figure 6A). Whole-atrial models presenting high G_K1_ and G_NaK_, and therefore high excitability (Figure 4), responded better to vernakalant and flecainide than to dronedarone. While none of them caused I_NaK_ or I_K1_ inhibition, the former yielded greater inward current block (Supplementary Table S2, Supplementary Figure S2). Thus, vernakalant- and flecainide-induced I_Na_ and I_CaL_ inhibition facilitated pharmacological cardioversion in those models presenting higher excitability.

### 3.6 Accuracy of prediction of AF cardioversion with specific treatments is 70% using both electrocardiogram metrics and ionic current profiles

**FIGURE 7:**
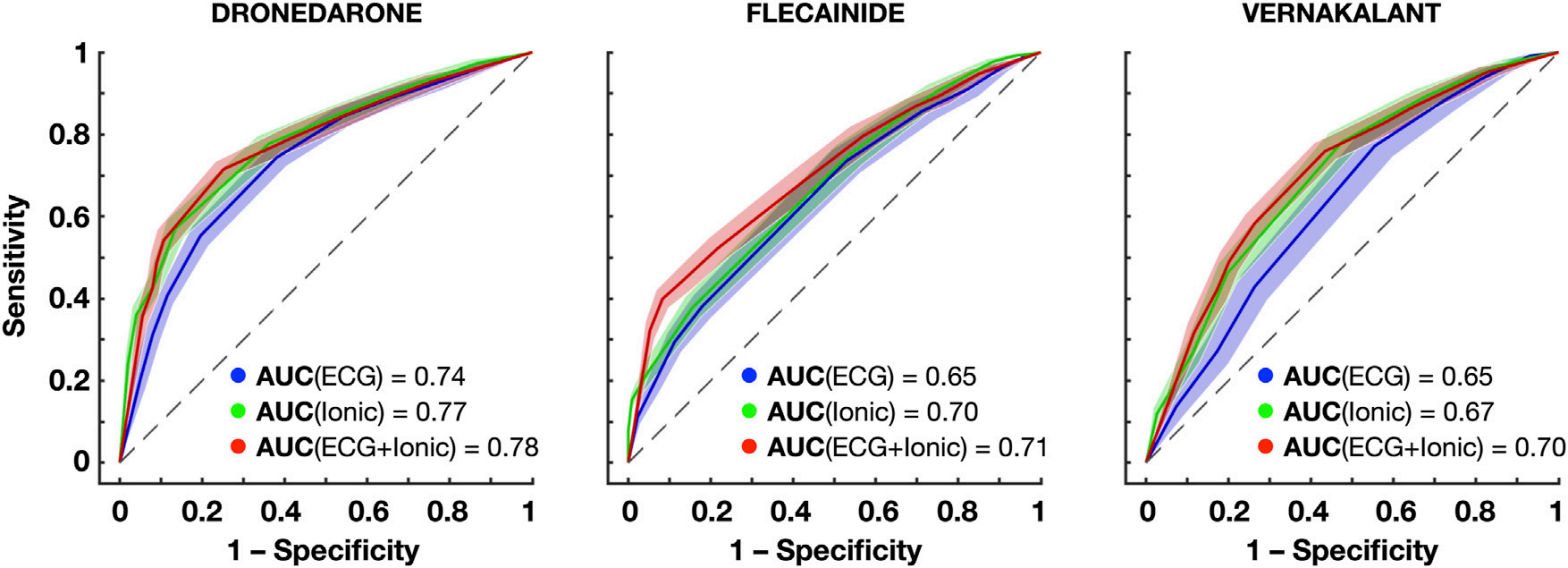
Receiver operating characteristics (ROC) curves for the prediction of successful cardioversion with dronedarone, flecainide and vernakalant using the ECG, ionic properties and both. The confidence interval around each ROC is obtained repeating 5-fold cross-validation 20 times. Abbreviations: AUC: Areas under the curve.

Clinical decision support systems were built based on the information of the atrial model sustaining AF (i.e., ionic current properties) and the ECG metrics obtained during AF. Figure 7 and Table 1 show the prediction accuracy and receiver operating characteristics (ROC) curves of successful cardioversion for the drugs illustrated in Figure 6.

Both the information of the ionic densities and the ECG biomarkers yielded high-accuracy predictions of successful AF cardioversion, with the former being slightly superior to the latter. Higher accuracies were obtained for dronedarone, due to a higher specificity (Figure 7). Dronedarone cardioverted around 50% of episodes, so that the dataset was more balanced (i.e., similar proportion of episodes cardioverted and not cardioverted). By contrast, the lowest specificity was obtained for vernakalant (70% cardioversion efficacy), with random oversampling slightly improving the prediction.

Three ionic densities were consistently selected as relevant features: G_K1_, G_Kur_, and G_NaK_. As illustrated in Figure 6, these ionic densities differed to a greater extent in atrial cardiomyocyte models that responded better to flecainide, vernakalant or dronedarone, and thus, were revealing of the most favorable treatment for each episode. Conversely, the dominant frequency, sample entropy and the relative harmonic energy (directly related to the dominant frequency) were selected as relevant ECG metrics, suggesting that AF dynamics also had a crucial role on cardioversion efficacy.

## 4 Discussion

Key features underlying AF inducibility, maintenance and pharmacological cardioversion are identified in a population of structurally-healthy atria with varying ionic current properties. In-silico trials for 10 antiarrhythmic treatments are consistent with previously-reported clinical trials, and identify mechanisms and predictors of successful cardioversion. Specific findings are: 1) AF inducibility in structurally-healthy atria might be explained by discordant APD alternans resulting from steep electrical restitution and high excitability (occurring for elevated L-type Ca^2+^ and inward rectifier K^+^ currents); 2) AF maintenance is also enabled by high excitability, for profiles with high inward rectifier K^+^ and Na^+^/K^+^ pump currents; 3) pharmacological cardioversion is optimal for treatments minimizing excitability, through maximal prolongation of refractoriness, which is dependent on individual ionic profiles; 4) successful pharmacological cardioversion is predicted in virtual human atria with >70% accuracy using both ionic current profiles or ECG metrics. Methodologically, our study demonstrates the power of in-silico drug trials conducted in human whole-atria models constructed based on hundreds of ionic current profiles, with perfect control over the parameters studied and high spatio-temporal resolution.

### 4.1 Precision medicine for atrial fibrillation management: *In-silico* drug trials

Currently, rhythm control therapy for AF follows a “one-size fits all” approach (Kany et al., 2021). However, the response of individual patients to rhythm control therapy is highly variable, and predicting its success is still a challenge (Kany et al., 2021). Previous studies have demonstrated that analyzing AF complexity through the ECG might help predicting flecainide cardioversion efficacy (Zeemering et al., 2018). Others assert that pharmacogenetics would bring the best tailored approach (Capucci et al., 2018). In our study, we have shown that pharmacological cardioversion can be predicted with over 70% accuracy using either ionic or ECG metrics in virtual human atria. This is important as it points towards the possibility of predicting the response of pharmacological treatment using both non-invasive markers and ionic current properties. Moreover, it highlights the integral part of multi-scale modeling and simulation for AF treatment personalization (Roney et al., 2022).

In our in-silico drug trial, G_K1_ was selected as one of the most informative properties for predicting AF cardioversion, together with G_NaK_ and G_Kur_. Inhibiting the Na^+^/K^+^ pump reduced the cellular excitability and hampered AF maintenance, consistent with its potential for rhythm control (Bueno-Orovio et al., 2014) and rate control strategy (i.e., digoxin; Hindricks et al., 2021). Similarly, the importance of the inward rectifier K^+^ current in regulating AF inducibility and maintenance has been proven by previous experimental and computational studies (Noujaim et al., 2007; Sánchez et al., 2017). Results obtained with the cellular model used, however, should be carefully interpreted, given the strong model-dependent effects. This is especially important when evaluating the antiarrhythmic efficacy of I_K1_ inhibition, since a higher sensitivity for I_K1_ block is reported for Courtemanche compared to other ionic models (Sánchez et al., 2014; Sutanto, 2022). Moreover, I_K1_ inhibition might have opposing effects on triggers and substrates. While effective in terminating fibrillatory activity, as shown in this study, blocking I_K1_ might promote triggered activity and focal excitations (Filgueiras-Rama et al., 2012). In this sense, safe restoration of sinus rhythm (i.e., absence of ventricular arrhythmias) resulted from I_K1_ inhibition in goats with persistent AF (Ji et al., 2017). Moreover, as in our simulations, higher cardioversion efficacies resulted from applying chloroquine (I_K1_ blocker, among others) than flecainide in sheep models (Filgueiras-Rama et al., 2012).

Flecainide cardioverted 56% of the simulated AF episodes, matching the efficacy observed in clinical trials with AF patients (Boriani et al., 1998; Reisinger et al., 2004). Compared to flecainide, a higher proportion of AF episodes (i.e., 65%) were terminated for vernakalant simulation. Similarly, a non-randomized retrospective study (Pohjantähti-Maaroos et al., 2019) observed higher number of patients cardioverted to sinus rhythm with vernakalant than flecainide (67 vs 46%, respectively).

Dronedarone also proved to be effective in terminating a great number of simulated AF episodes. Dronedarone has shown a positive impact on clinical endpoints for paroxysmal AF patients and compared to amiodarone, it delays and reduces AF occurrence (Singh et al., 2008). However, amiodarone has proven more effective than dronedarone in rhythm control (Hindricks et al., 2021).

In our study, amiodarone showed the lowest cardioversion efficacy. Nevertheless, cardioversion rates as high as 87% are observed after 24 h from its administration (Bash et al., 2012). Amiodarone inhibits multiple ionic channels, including I_CaL_ and I_Na_. Blocking both channels reduces cellular excitability (Loewe et al., 2014; Liberos et al., 2016) and amiodarone-induced I_Na_ inhibition has been suggested as the main mechanism underlying rotor termination (Wilhelms et al., 2013). In our study, however, the baseline atrial cardiomyocyte models yielding AF presented increased G_Na_ and G_CaL_ compared to the baseline model. Consequently, the inward current block induced by amiodarone was not sufficient to reduce excitability and terminate AF. This is in agreement with previous simulations studies, that showed that the antiarrhythmic effects of I_Na_ and I_CaL_ block depended on their basal ionic densities (Liberos et al., 2016). Of note, amiodarone is primarily recommended in the context of severe heart failure (Andrade et al., 2020), in which I_CaL_ is expected to be down-regulated (Denham et al., 2018). Thus, amiodarone-induced I_CaL_ inhibition might have a lower impact on the APD and higher on excitability than in our study. It has been also suggested that amiodarone metabolites, such as desethylamiodarone, which accumulates after long-term therapy with amiodarone, might be the actual responsible for the increased atrial refractory period (Talajic, DeRoode and Nattel, 1987).

Furthermore, amiodarone-induced I_CaL_ inhibition flattened the APDR curve, reduced the frequency and amplitude of APD alternans and prevented AF inducibility in the structurally-healthy atria. Therefore, while ineffective for AF termination, amiodarone hindered the formation of dynamic substrates derived from steep electrical restitution.

### 4.2 Ionic current dysregulation and dynamic electrophysiological substrates in structurally-healthy atria

Steep electrical restitution was the mechanism adopted in this study to trigger dynamic substrates that facilitate AF, as observed in human AF patients (Narayan et al., 2008; Krummen et al., 2012). Unlike previous simulation works that changed single ionic currents (Garfinkel et al., 2000), steep electrical restitution in our study resulted from specific multivariate ionic density distributions. These distributions were obtained by scaling uniformly all ionic densities of the cellular model, which mimicked inter-patient variability in ionic densities and preserved the high non-linearity between ion channel conductances and the action potential morphology (Elliott et al., 2021). Moreover, we considered a detailed atrial geometry with realistic anisotropy ratios and CV, as opposed to using monolayers (Virag et al., 2002) or isotropic conduction (Gong et al., 2007), and replicate accurate AF-initiation conditions.

As such, we observed that increased G_CaL_, G_K1_ and G_NaK_ characterized those atrial cardiomyocyte models favoring AF inducibility and maintenance in structurally-healthy atria. Previous simulation studies observed that increasing G_CaL_ steepens the APD restitution slope, potentially leading to wave break-up and fibrillation (Garfinkel et al., 2000). In our study, however, we found that steep restitution was only engaged for high cellular excitability. The latter was enabled by G_K1_ up-regulation, and could be aggravated by increased acetylcholine-activated inward rectifier K^+^ (I_K,ACh_) current, not considered in the present study.

Likewise, elevated G_K1_ and G_NaK_ ensured AF maintenance. In agreement with our simulations, increased Na^+^/K^+^ pump was linked to higher susceptibility to postoperative AF (Tran et al., 2009). Increased inward rectifier K^+^ current has been extensively associated with the AF-induced electrophysiological remodeling (Dobrev and Ravens, 2003) and proven essential for rotor stabilization in the electrically remodeled atria (Pandit et al., 2005). In our population of structurally-healthy atria, however, high G_K1_ was additionally required for AF inducibility. Similarly, other computational studies have observed that in the absence of fibrosis, the inhibition of the inward rectifier K^+^ current considerably hindered AF initiation (Gharaviri et al., 2021). A genetic analysis performed in a population of kindreds (Xia et al., 2005) identified a gain-of-function mutation in KCNJ2, encoding the Kir2. x channels of the inward rectifier K^+^ current, underlying familial AF. Missense mutations in KCNJ2 were also reported in human patients with paroxysmal AF (Deo et al., 2013). Therefore, as shown in this study, increased inward rectifier K^+^ current constitutes an AF predisposing condition that additionally favors AF perpetuation.

### 4.3 Limitations and future perspectives

Drug action was simulated as simple pore-block models as the data required were available for the compounds investigated. However, it is possible that this might have increased the model sensitivity to repolarization prolonging effects, explaining the grater efficacy of class III antiarrhythmic drugs compared to class Ic. Neglecting the rate dependence of class Ic agents and thus, potentially underestimating I_Na_ inhibition, might explain the different results obtained for propafenone and flecainide. Similarly, increasing the resting membrane potential through I_K1_ inhibition, therefore hampering I_Na_ (in)activation, resulted more effective than direct I_Na_ block. This might reflect the importance of class Ic agents for binding with different affinities to the different states of the Na^+^ channel, which was not considered in this study.

Moreover, pharmacological treatment and the performance of the predictive model should be further evaluated considering, not only electrophysiological, but also anatomical and structural variability. Even the electrophysiological variability considered was small. The 97 models selected, which presented the ionic current properties required to induce AF in structurally-healthy atria, had similar morphologies (i.e., a rapid phase 3 repolarization and relatively hyperpolarized resting membrane potential). Therefore, a wider range of action potentials has to be taken into account in future studies.

Restricting the study to similar action potential morphologies could also explain why the accuracy of the clinical decision support system was not higher than 70%. A better stratification of the ionic current properties that respond to specific antiarrhythmic drugs would result from considering a wider ionic current distribution in the first place (i.e., wider range of action potential morphologies). The latter could improve the prediction accuracy and boost associated prediction metrics. In this sense, employing other machine learning algorithms might also add extra predictive power.

In the clinic, obtaining the ionic density of the patient presents a challenge, especially without undertaking an invasive procedure (i.e., biopsy during surgery). However, the finding that similar accuracies are obtained with the ECG and ionic metrics for predicting successful AF cardioversion, suggests that the ECG already contain information of the ionic current properties. Thus, further studies should investigate whether the atrial ionic profile of a patient could be characterized non-invasively through the ECG.

## 5 Conclusion

In structurally-healthy atria, AF inducibility and sustainability are enabled by high excitability and steep restitution, due to elevated L-type Ca^2+^ and inward rectifier K^+^ currents. Accordingly, decreasing excitability, through prolonged refractoriness, results in pharmacological cardioversion. However, maximal prolongation of refractoriness depends on the interaction between the ionic distribution of the atria and the ionic currents targeted by the antiarrhythmic drug, highlighting optimal pharmacological treatments for individual ionic current profiles. Therefore, successful cardioversion is predicted in virtual human atria with 70% accuracy from both ionic current and ECG properties.

## Data Availability

The original contributions presented in the study are included in the article/Supplementary Material, further inquiries can be directed to the corresponding authors.
