# Supplementary material for: In-silico drug trials for precision medicine in atrial fibrillation: From ionic mechanisms to electrocardiogram-based predictions in structurally-healthy human atria
Source: Front Physiol. 2022 Sep 15;13:966046. doi: 10.3389/fphys.2022.966046 (PMC9522526; doi:10.3389/fphys.2022.966046)
Supplement: Supplementary file 1 [file Table1.DOCX]

Supplementary Material

# Supplementary Methods

## Population of human atrial cardiomyocyte models

Twelve ionic current conductances and permeabilities were sampled in the population of atrial cardiomyocyte models: The ultrarapid, rapid and slow delayed-rectifier K^+^ current density (G_Kur_, G_Kr_ and G_Ks_), transient outward K^+^ current density (G_to_), inward rectifier K^+^ current density (G_K1_), L-type Ca^2+^ current density (G_CaL_), fast Na^+^ current density (G_Na_), Na^+^/K^+^ pump (G_NaK_), Ca^2+^/Na^+^ exchanger (G_NCX_) and the permeabilities of the sarcoplasmic reticulum Ca^2+^ release (G_rel_), leak (G_leak_) and uptake (G_up_) currents.

Inter-patient variability resulting from scaling the above-mentioned conductances was characterized by the following action potential (AP) biomarkers: AP duration (APD) at 20%, 50% and 90% of cellular repolarization (APD_20_, APD_50_ and APD_90_, respectively), AP amplitude (APA), resting membrane potential (RMP) and maximum upstroke velocity (dV/dt_max_). AP triangulation, computed as APD_90_–APD_50_ (Muszkiewicz *et al.*, 2018), was used to measure the prolongation of the plateau phase in cardiomyocyte models presenting steep APD restitution (APDR).

## Action potential duration and conduction velocity restitution

APDR was obtained by both standard S1S2 and dynamic protocols. The standard S1S2 protocol was computed in a single-cell environment and consisted of 50 S1 stimuli applied at a specific cycle length (CL) followed by an extra S2 stimulus each time at decreasing CL. Six different CLs (700, 600, 500, 400, 300 and 250 ms) were employed for the S1 stimuli. The S2 stimulus started at the same CL as the last S1 and was decreased by 50 ms from 700 to 400 ms, by 20 ms from 400 to 300 ms, and by 10 ms from 300 ms until the previous diastolic interval (DI) was lower than zero.

The dynamic restitution protocol and conduction velocity (CV) restitution (CVR) were computed using an isotropic cable (40000×100×100 μm) as in Wilhelms *et al.*, 2013. In both cases, five consecutive beats were paced at one side of the cable at different CLs, following the same interval progression as for the standard S1S2 protocol. Cardiomyocyte models were previously paced for 50 beats in a single-cell environment, so they could adapt to the different CLs. The CV was computed dividing the distance travelled by the wavefront by the time at which the cell located at three-quarters down the cable reached the maximum depolarization upstroke. The APDR was computed recording the last two APD and DIs in that same cell to detect transient alternation. APDR and CVR were calculated only if all five beats propagated in the cable.

The diffusion coefficient was adjusted to obtain a CV of 60 cm/s (Sánchez *et al.*, 2017; **Suppl. Table 1**).

## Whole-atria models with homogeneous vs. heterogeneous ionic properties

Every single-cell model presenting steep APDR and CVR was used to populate a human-based whole-atria model with homogeneous and heterogeneous ionic properties. For the homogeneous population, one atrial cardiomyocyte model was included in all atrial regions. For the heterogeneous electrophysiology, the cardiomyocyte model was included at the left atrium and modified elsewhere (**Suppl. Figure 1**). The specific electrophysiological changes are summarized in **Suppl. Table 1**.

**Supplementary Figure 1.** Population of whole-atria models with homogeneous and heterogeneous ionic properties. Abbreviations. **RA-LA:** Right and left atrium; **RAA-LAA:** Right and left atrial appendage; **CT:** Crista terminalis; **PM:** Pectinate muscles; **AVR:** Atrio-ventricular rings (mitral and tricuspid valves). **SCV:** Superior cava vein. **rPV:** Right pulmonary veins.

**Supplementary Table 1.** Tissue anisotropy and ionic heterogeneities. Anisotropy and CV differences were applied in both populations of whole-atria models, homogeneous and heterogeneous. The ionic current modification (multiplicative factors with respect to the baseline model) only in the population with heterogeneous ionic properties. Abbreviations as in Suppl. Figure 1. **BB:** Bachmann’s Bundle. **SAN:** Sinoatrial node. Adapted from Sánchez *et al.*, 2017.

|  | Anisotropic ratio  Transversal:Longitudinal | CV (cm/s) | I_to_ | I_CaL_ | I_Kr_ |
| --- | --- | --- | --- | --- | --- |
| LA | 1:2 | 59 | Baseline cardiomyocyte model | | |
| RA | 1:2 | 59 | 1 | 1 | 1/1.6 |
| SAN | 1:1 | 31 | 1 | 1 | 1 |
| SAN | 1:1 | 31 | 1 | 1 | 1 |
| CT | 1:10 | 116 | 1.35 | 1.6 | 0.9 |
| PM | 1:2 | 98 | 1.05 | 0.95 | 0.9 |
| BB | 1:2 | 98 | 1 | 1 | 1 |
| LAA | 1:2 | 59 | 0.65 | 1.05 | 2.75 |
| AVR | 1:2 | 59 | 1.05 | 0.65 | 3 |

## In-silico drug modeling and simulation

Eight antiarrhythmic drugs were modelled according to their 50% inhibitory concentration (IC_50_), hill coefficient (h) and effective plasma drug concentration. These values were extracted from previous modelling studies, resulting in highly variable formulations depending on the study considered (**Suppl. Table 2**). In order to overcome this variability, simulations were repeated with different drug formulations. Therefore, if one drug was modelled differently in previous studies, or presented different parameters (i.e., IC_50_, h and concentration) all combinations were tested in this study. Accordingly, some drugs were simulated different times using different formulations, different drug concentrations or both (**Suppl. Table 2**, **Suppl. Figure 2**). The percentage of ionic current block resulting from simulating the eight antiarrhythmic drugs, with different IC_50_, h and drug concentrations, is illustrated in **Suppl. Figure 2**.

Only one formulation is displayed in the figures of the main paper (i.e., that with the highest cardioversion efficacy). This is highlighted in **Suppl. Table 2** by “This study” written in bold letters.

Evaluation of simulated drug action against experimental data can be found in the **Suppl. Table 3**.

**Supplementary Table 2.** List of antiarrhythmic drugs with their IC_50_ (μM) and hill coefficient (in brackets). The drug concentration used is presented in brackets next to the study. Comparison between the modelling approach adopted by previous and the present study.

| **Drug** |  | **Ionic current IC50 (h)** | | | | | | | | |
| --- | --- | --- | --- | --- | --- | --- | --- | --- | --- | --- |
|  | **Study** | **INa** | **Ito** | **ICaL** | **IKur** | **IKr** | **IKs** | **IK1** | **INCX** | **INaK** |
| **Amiodarone** | (Loewe *et al.*, 2014) (**2.33 μM**) | 4.84 (0.76) | – | 5.80 (1.0) | – | 2.8 (0.91) | 3.84 (0.63) | – | 3.3 (1.0) | 15.6 (1.0) |
|  | (Sutanto *et al.*, 2019) (**10 μM**) | 5 (1.0) | 3.8 (0.4) | 1.5 (0.6) | – | 3.0 (1.0) | 100 (1.0) | – | 3.4 (1.0) | – |
|  | (Bai et al., 2020) (**1.55** **μM**) | 4.84 (0.76) | 4.90 (1.0) | 0.4 – 5.8 (1.0) | 132.86 (1.0) | 10 (1.0) | 3.84 (0.63) | – | 3.6 (1.0) | 15.6 (1.0) |
|  | This study (2.0 μM) | 4.84 (0.76) | 4.9 (1.0) | 5.80 (1.0) | – | 2.8 (1.0) | 3.84 (0.63) | – | 3.3 (1.0) | 15.6 (1.0) |
|  | **This study (2.0 μM)** | 5 (1.0) | 3.8 (0.4) | 1.5 (0.6) | – | 3.0 (1.0) | 100 (1.0) | – | 3.4 (1.0) | – |
| **Dronedarone** | (Loewe *et al.*, 2014) (**0.205** **μM**) | 0.54 (2.03) | – | 0.83 (2.75) | 1.0 (1.0) | 0.0591 (0.8) | 5.6 (0.51) | – | – | – |
|  | (Sutanto *et al.*, 2019) (**10 μM**) | 1 (1.7) | – | 4 (1.0) | – | 2 (1.0) | 10 (1.0) | – | – | – |
|  | This study (1.0 μM) | 1 (1.7) | – | 4 (1.0) | – | 2 (1.0) | 10 (1.0) | – | – | – |
|  | **This study (0.205 μM)** | 0.54 (2.03) | – | 0.83 (2.75) | 1.0 (1.0) | 0.0591 (0.8) | 5.6 (0.51) | – | – | – |
| **Flecainide** | (Sutanto *et al.*, 2019) (**3 μM**) | 6.5 (1.0) | 10.0 (0.8) | 27.1 (1.0) | 2.9 (1.0) | 1.6 (1.0) | – | – | – | – |
|  | - (Bai *et al.*, 2020) (**2 μM**) | 84 (1.0) | – | – | – | 1.5 (1.0) | – | – | – | – |
|  | **This study (2.5 μM)** | 6.5 (1.0) | 10.0 (0.8) | 27.1 (1.0) | 2.9 (1.0) | 1.6 (1.0) | – | – | – | – |
| **Propafenone** | (Sutanto *et al.*, 2019) (**1.5 μM**) | 2.5 (1.0) | 7.2 (1.0) | 1.55 (1.0) | – | 0.44 (1.0) | 16 (1.0) | – | – | – |
|  | (Bai *et al.*, 2021) (**0.2–0.8 μM**) | 1.2 (1.0) | 4.8 (1.0) | 1.7 (1.0) | 4.4 (1.0) | 2.0 (1.0) | – | 16.8 (1.0) | – | – |
|  | This study (0.8 μM) | 1.2 (1.0) | 4.8 (1.0) | 1.7 (1.0) | 4.4 (1.0) | 2.0 (1.0) | – | 16.8 (1.0) | – | – |
|  | **This study (0.8 μM)** | 2.5 (1.0) | 7.2 (1.0) | 1.55 (1.0) | – | 0.44 (1.0) | 16 (1.0) | – | – | – |
| **Ranolazine** | (Sutanto *et al.*, 2019) (**10 μM**) | 200 (1.0) | – | 250 (1.0) | – | 12 (1.0) | 100 (1.0) | – | 91 (1.0) | – |
|  | **This study (10 μM)** | 200 (1.0) | – | 250 (1.0) | – | 12 (1.0) | 100 (1.0) | – | 91 (1.0) | – |
| **Vernakalant** | (Sutanto *et al.*, 2019) (**30 μM**) | 90 (1.0) | 15 (1.0) | 84 (1.0) | 15 (1.0) | 20 (1.0) | – | – | – | – |
|  | (Loewe *et al.*, 2015) **(30 μM)** | 15.07 (0.95) | 30 (0.82) | 84 (1.0) | 13 (0.92) | 21 (0.92) | – | – | – | – |
|  | This study (30 μM) | 90 (1.0) | 15 (1.0) | 84 (1.0) | 15 (1.0) | 20 (1.0) | – | – | – | – |
|  | **This study (10 μM)** | 90 (1.0) | 15 (1.0) | 84 (1.0) | 15 (1.0) | 20 (1.0) | – | – | – | – |
|  | This study (30 μM) | 15.07 (0.95) | 30 (0.82) | 84 (1.0) | 13 (0.92) | 21 (0.92) | – | – | – | – |
| **Ibutilide** | (Sutanto *et al.*, 2019) (**0.01 μM**) | – | – | – | – | 0.02 (1.0) | – | – | – | – |
|  | This study (0.01 μM) | – | – | – | – | 0.02 (1.0) | – | – | – | – |
|  | **This study (0.015 μM)** | – | – | – | – | 0.02 (1.0) | – | – | – | – |
| **Digoxin** | (Bai, Lu and Zhang, 2020) (**1 nM**) | – | – | – | – | 0.054 (1.0) | – | – | – | 0.12 (1.0) |
|  | **This study (0.04 μM)** | – | – | – | – | 0.054 (1.0) | – | – | – | 0.12 (1.0) |
|  | This study (0.05 μM) | – | – | – | – | 0.054 (1.0) | – | – | – | 0.12 (1.0) |

**Supplementary Figure 2.** Ionic current block (%) exerted by the antiarrhythmic drugs modelled in this study. Order of appearance as listed in Suppl. Table 2.

**Supplementary Table 3.** Comparison of the rate-dependent effects of different drugs in-silico (labelled as “This study”) and in-vitro (previously published). The in-silico results are obtained from the single-cell models leading to sustained AF, before (“Control”) and after drug modelling. For the in-vitro results, data are presented in parentheses if the authors reported the dose-dependent effects of a drug. The symbol (*) is added in those cases that the cycle length was not exactly the one considered in this table but similar. In both in-silico and in-vitro, data are presented as mean ± standard deviation. If the standard deviation is not reported in-vitro, data were not available.

| **Control** | **Study** |  | **APD (ms)** | | | **CV (cm/s)** | | | **Vmax (mV/ms)** | | |
| --- | --- | --- | --- | --- | --- | --- | --- | --- | --- | --- | --- |
|  |  | Cycle Length (ms) | **1000** | **500** | **300** | **1000** | **500** | **300** | **1000** | **500** | **300** |
|  | This study |  | 276 ± 31 | 252 ± 35 | 199 ± 25 | 63 ± 5 | 63 ± 5 | 60 ± 6 | 211± 3 | 208 ± 30 | 162 ± 50 |

| **Drug** | **Study** | **Species** | **APD Change (%)** | | | **CV Change (%)** | | | **Vmax Change (%)** | | |
| --- | --- | --- | --- | --- | --- | --- | --- | --- | --- | --- | --- |
|  |  |  | **1000** | **500** | **300** | **1000** | **500** | **300** | **1000** | **500** | **300** |
| **Amiodarone** | This study |  | -28 ± 13 | -29 ± 6 | -18 ± 7 | -12 ± 1 | -13 ± 1 | -6 ± 11 | -21 ± 1 | -21 ± 1 | -13 ± 5 |
|  | (Quinteiro et al. 1994)  (Sun et al., 2002)  (Suzuki *et al.*, 2013) | Dog  Rabbit  Rabbit | –  -11  -2 | –  -11  – | –  -8  – | -2  –  – | -3  –  – | -5  –  -19 ± 4 | -4  -7  – | -11  -8  – | -21  -8  – |
| **Dronedarone** | This study |  | 30 ± 8 | 19 ± 12 | 28 ± 23 | -5 ± 1 | -5 ± 2 | -5 ± 3 | -9 ± 1 | -12 ± 4 | -13 ± 6 |
|  | (Sun et al. 1999)  (Sun et al. 2002) | Rabbit  Rabbit | [27, 30]*  15 | [15, 20]*  15 | [5, 12]  17 | – | – | – | –  -6 | –  -7 | –  -6 |
| **Flecainide** | This study |  | 21 ± 14 | 22 ± 9 | 29 ± 14 | -13 ± 1 | -12 ± 1 | -16 ± 4 | -23 ± 1 | -24 ± 2 | -65 ± 30 |
|  | (Wang *et al.*, 1990)  (O’Hara *et al.*, 1992)  (Hatem *et al.*, 1992)  (Wang et al. 1995)  (Van Hunnik *et al.*, 2016) | Human  Dog  Human Dog  Goat | 6 ± 3  –  12*  –  – | –  [5 , 20]  –  –  – | 27 ± 12  [7, 39]  –  –  – | –  –  –  –  – | –  –  –  - 8*  [-12, -24]* | –  –  –  - 11  [-16, -33]* | - 28  –  - 21*  –  – | –  –  –  –  – | -50  –  –  –  – |
| **Propafenone** | This study |  | 0 ± 21 | -9 ± 12 | -7 ± 9 | -10 ± 1 | -10 ± 1 | 5 ± 9 | -19 ± 1 | -19 ± 1 | -32 ± 50 |
|  | (Dukes et al. 1984)  (Wang *et al.*, 1993)  Burashnikov et al., 2012a | Rabbit  Dog  Dog | –  –  – | [-1, 24]  –  [0, 2] | –  –  [-2, 0] | –  – | [-15, -24]  – | –  -29 ± 4 | –  – | [-7, -77]  –  [-15, -41] | –  –  [-30, -77] |
| **Ranolazine** | This study |  | 12 ± 7 | 6 ± 6 | 4 ± 8 | -2 ± 1 | -2 ± 1 | -1 ± 5 | -3 ± 1 | -4 ± 1 | -17 ±7 |
|  | (Burashnikov *et al.*, 2007)  (Burashnikov et al. 2012a)  (Burashnikov et al. 2012b) | Dog  Dog  Dog | –  –  7 | 8  9  5 | –  11  8 | –  –  – | -16  –  – | -35  –  – | –  –  – | -24 ± 5  -26 ± 6  -23 ± 13 | –  -57 ± 17  -48 ± 18 |
| **Vernakalant** | This study |  | 6 ± 13 | 11 ± 13 | 11 ± 11 | -5 ± 1 | -4 ± 1 | -6 ± 6 | -7 ± 1 | -8 ± 1 | -15 ± 34 |
|  | (Burashnikov *et al.*, 2012)  (Wettwer *et al.*, 2013)  (Van Hunnik *et al.*, 2016) | Dog  Human  Goat | 9  7  – | 7  –  – | 4  11  – | –  –  – | –  –  [-15, -18] | –  –  [-15, -21] | –  -23 ± 7  – | -20 ± 12  –  – | -22 ± 8  -32 ± 5  – |
| **Ibutilide** | This study |  | 13 ± 5 | 10 ± 7 | 12 ± 7 | 0 | 0 | 0 | 0 ± 1 | 0 | 0 |
|  | (Lee, 1992)  (Lee and Lee, 1998)  (Oshikawa *et al.*, 2001) | Guinea pigs  Human  Human | –  [4, 19]  – | [5, 39]*  –  23* | –  –  24* | –  –  – | –  –  – | –  –  – | –  –  – | –  –  – | –  –  – |
| **Digoxin** | This study |  | 19 ± 9 | 21 ± 8 | 26 ± 6 | 0 | 0 | -5 ± 8 | -2 ± 1 | -3 ± 3 | -44 ± 76 |
|  | (Wang *et al.*, 2007) | Guinea pig | [22, 100]* | – | – | – | – | – | – | – | – |

## Predictive models

Three datasets were built combining the information obtained from the simulated 8-lead ECG, action potential shape, and ionic current densities. The first dataset comprised five biomarkers (i.e., dominant frequency, organization index, Shannon’s spectral entropy, sample entropy, and relative harmonic energy) obtained for each lead of the simulated 8-lead ECG, yielding 40 biomarkers. The second dataset included the ionic properties of the model, namely baseline APD_90_, CV, RMP and the 12 ionic densities describing the electrophysiology of the whole-atria model. The third dataset combined the information of both datasets, ECG metrics and ionic properties.

The output vector consisted of a binary array of 1s, describing the simulated AF episodes cardioverted by a drug, and 0s, otherwise. Therefore, for every drug studied, a binomial classification was predicted by logistic regression (Zeemering *et al.*, 2018). A detailed explanation can be found in Zeemering *et al.*, 2018. Briefly, an elastic net logistic regression was built with the updated version of the Glmnet toolbox (available http://hastie.su.domains/glmnet_matlab/), available for MATLAB (MATLAB, 2020b release, The MathWorks, Inc., Natick, MA, USA). Parameter selection was done by stepwise logistic regression using alpha equal 0.5 and 100 values for lambda. The performance of the predictive models was validated through 5-fold cross-validation, repeated 20 times with random subsampling. Receiver Operating Characteristics (ROC) curves were obtained for all three datasets.

# Results

## Discordant APD alternans and AF inducibility

**Suppl. Figure 3** (panel A, left) illustrates the local activation times (LAT) map and sequence of activation during sinus rhythm (SR). The transmembrane voltage (V_m_) is recorded at three sites of the posterior wall of the left atrium during the last SR beat and first two S2 stimuli in the burst (panel A, right). “Panel B, top” shows the APD alternans map obtained subtracting the APD of the last SR beat to the APD of the first S2 stimulus. The identity line (white) separates regions alternating in a short-long pattern (green) from those showing a long-short APD alternation (purple). “Panel B, bottom” depicts V_m_ snapshots following the second S2 stimulus, highlighting conduction block and re-entry.

During SR, the wavefront traveled from the right pulmonary veins, throughout the posterior wall, to the left pulmonary veins, as illustrated through the green arrow and LAT map in panel A. The application of the burst pacing inverted the sequence of activation (red arrow) and the left pulmonary veins were firstly depolarized. Since the left pulmonary veins were lastly depolarized during SR and the first during ectopic stimulation, a very short DI arose (panel A, red V_m_). This, in turn, yielded a very short APD, so that regions near the left pulmonary veins alternated with a long-short pattern. Following the same reasoning, regions close to the right pulmonary veins presented a very long DI, which led to a very long APD (panel A, yellow V_m_). These regions alternated with a short-long pattern. Along both regions, a third region presented the same APD during the first S2 stimulus than during SR (panel A, orange V_m_).

This led to discordant alternans, characterized by regions alternating with opposing APD phase. In this scenario, when the following S2 stimuli were applied, only those regions that presented short APD (panel B, purple and red dot) were able to propagate the wavefront. Conduction block (green star) arose in regions with long APD (panel B, purple and yellow dot), yielding re-entry and AF initiation. The steep restitution properties that favored APD alternans, conduction block and re-entry, additionally facilitated AF maintenance after AF inducibility.

**Supplementary Figure 3.** Discordant alternants favoring atrial fibrillation inducibility. **A)** Local activation times (LAT) during sinus rhythm and time course of the transmembrane voltage (V_m_) at three sites of the posterior wall of the left atrium. The application of the burst pacing inverts the activation sequence at the latter. The green arrow represents the direction of wavefront propagation during sinus rhythm (SR) and the red one during burst pacing. **B)** Action potential duration alternans map, showing discordant alternation: purple regards long-short alternation and green short-long alternation. The color dots correspond to the representative action potential traces shown in A), illustrating short-long alternation (yellow dot), long-short alternation (red dot) and no alternation (orange dot placed at the line of identity). Snapshots of V_m_ following the second S2 stimuli in the burst. The red star represents the location of the burst pacing and the green star conduction block.

## Imbalanced Ca^+2^ handling and high-magnitude APD alternans

**Suppl. Figure 4** shows the APDR curves of AF-inducible and non-inducible models (Suppl. Figure 4, top; as in **Figure 3**, Manuscript), AF-inducible models with elevated and control L-type Ca^2+^ current (Suppl. Figure 4, middle) and inward rectifier K+ current (Suppl. Figure 4, bottom). The APDR curves are plotted against the CLs used in the burst pacing.

AF-inducible models presented greater magnitude of APD alternans compared to non-inducible models (67.3±92.5 vs. 8.9±6.6 ms, AF-inducible vs. non-inducible models) (Suppl. Figure 4, top). Restoring the baseline inward rectifier K^+^, and especially L-type Ca^2+^ current, in AF-inducible decreased the magnitude of APD alternans (67.3±92.5 vs. 8.3±6.0 ms, AF-inducible models with elevated vs. control L-type Ca^2+^ current; 67.3±92.5 vs. 18.2±12.1; AF-inducible models with elevated vs. control inward rectifier K^+^ current). Reducing the inward rectifier K^+^, but not the L-type Ca^2+^ current, significantly decreased the number of models propagating at short CLs.

**Supplementary Figure 4.** Dynamic action potential duration (APD) restitution curve plotted against the cycle lengths used in the burst pacing ([170, 260] ms). Comparison between AF-inducible and non-inducible models (top), AF-inducible models with elevated and control L-type Ca^2+^ current (middle) and AF-inducible models with elevated and control inward rectifier K^+^ current (middle).

## Comparison of in-silico drug effects between different cellular models

**Suppl. Figure 5** illustrates a comparison of APD variation (%) after drug action between the CRN, Grandi-Bers (GB) (Grandi *et al.*, 2011) and Maleckar-Trayanova (MT) (Maleckar *et al.*, 2009) models. Although similar trends were observed for the antiarrhythmic drugs tested in all three ionic models, the GB and MT models were more sensitive to I_Kur_ inhibition than the CRN model (Suppl. Figure 5; vernakalant). On the other hand, the CRN model showed a greater APD prolongation after I_Kr_ inhibition compared to the GB and MT models (Suppl. Figure 6; dronedarone, ibutilide). This is due to a predominant contribution of I_Kur_ on the cellular repolarization of the latter, with neither I_Kr_ nor I_Ks_ playing a major role (Maleckar *et al.*, 2009).

**Supplementary Figure 5.** Comparison of APD variation (%) after drug action between the Courtemanche-Ramirez-Nattel (CRN 1998), Grandi-Bers (GB 2011) and Maleckar-Trayanova (MT 2009) models. The ionic profiles tested are the same as in Suppl. Figure 6 and Figure 5, Manuscript.

**Suppl. Figure 6** illustrates the dynamic APDR curve and shortest CL enabling propagation (i.e., ERP) prior and after drug action. These biomarkers have been computed for the atrial cardiomyocyte models yielding sustained AF in the population with homogeneous and heterogeneous ionic properties.

**Supplementary Figure 6.** **A)** Action potential duration (APD) restitution in control conditions (“No drug”) and after drug modelling for the atrial cardiomyocyte models yielding sustained atrial fibrillation in the population of whole-atria models with homogeneous and heterogeneous ionic properties. **B)** Shortest cycle length (ms) enabling propagation (i.e., effective refractory period, ERP)

**Suppl. Figure 7** and **8** show consecutives snapshots of a representative AF episode and the ECG, respectively, in control conditions, after amiodarone application and I_K1_ inhibition.


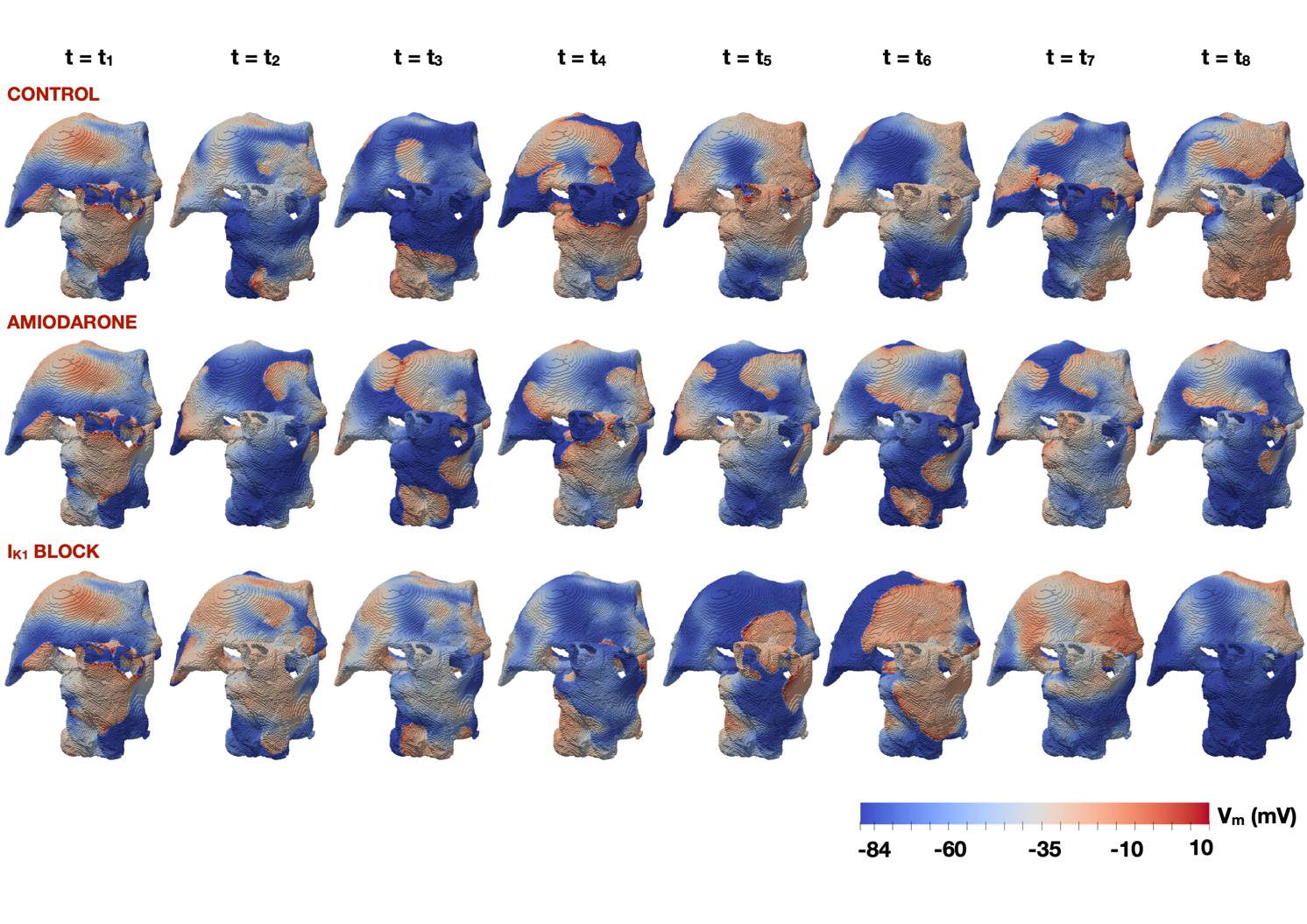


**Supplementary Figure 7.** Consecutive snapshots of a representative AF episode in control, after amiodarone application and I_K1_ inhibition.

**Supplementary Figure 8.** Simulated electrocardiogram (ECG – Lead V4) for the three episodes illustrated in Suppl. Figure 6.

# References

Bai, J. *et al.* (2020) ‘In silico investigation of the mechanisms underlying atrial fibrillation due to impaired Pitx2’, *PLoS Computational Biology*, 16(2). doi: 10.1371/journal.pcbi.1007678.

Bai, J. *et al.* (2021) ‘In silico assessment of class I antiarrhythmic drug effects on pitx2‐induced atrial fibrillation: Insights from populations of electrophysiological models of human atrial cells and tissues’, *International Journal of Molecular Sciences*, 22(3), pp. 1–30. doi: 10.3390/ijms22031265.

Bai, J., Lu, Y. and Zhang, H. (2020) ‘In silico study of the effects of anti-arrhythmic drug treatment on sinoatrial node function for patients with atrial fibrillation’, *Scientific Reports*, 10(1), pp. 1–15. doi: 10.1038/s41598-019-57246-5.

Burashnikov, A. *et al.* (2007) ‘Atrium-selective sodium channel block as a strategy for suppression of atrial fibrillation: Differences in sodium channel inactivation between atria and ventricles and the role of ranolazine’, *Circulation*, 116(13), pp. 1449–1457. doi: 10.1161/CIRCULATIONAHA.107.704890.

Burashnikov, A. *et al.* (2012) ‘Rate-dependent effects of vernakalant in the isolated non-remodeled canine left atria are primarily due to block of the sodium channel comparison with ranolazine and dl-sotalol’, *Circulation: Arrhythmia and Electrophysiology*, 5(2), pp. 400–408. doi: 10.1161/CIRCEP.111.968305.

Burashnikov, A., Belardinelli, L. and Antzelevitch, C. (2012) ‘Atrial-selective sodium channel block strategy to suppress atrial fibrillation: Ranolazine versus propafenone’, *Journal of Pharmacology and Experimental Therapeutics*, 340(1), pp. 161–168. doi: 10.1124/jpet.111.186395.

Dukes, I. D. and Williams, E. m. V. (1984) ‘The multiple modes of action of propafenone’, *European Heart Journal*, 5(2), pp. 115–125. doi: 10.1093/oxfordjournals.eurheartj.a061621.

Grandi, E. *et al.* (2011) ‘Human Atrial Action Potential and Ca2+ Model: Sinus Rhythm and Chronic Atrial Fibrillation’, *Circulation Research*, 109(9), pp. 1055–1066. doi: 10.1161/CIRCRESAHA.111.253955.Human.

Hatem, S. *et al.* (1992) ‘Differential effects of quinidine and flecainide on plateau duration of human atrial action potential’, *Basic Research in Cardiology*, 87(6), pp. 600–609. doi: 10.1007/BF00788670.

Van Hunnik, A. *et al.* (2016) ‘Antiarrhythmic effect of vernakalant in electrically remodeled goat atria is caused by slowing of conduction and prolongation of postrepolarization refractoriness’, *Heart Rhythm*. Elsevier, 13(4), pp. 964–972. doi: 10.1016/j.hrthm.2015.12.009.

Lee, K. S. (1992) ‘Ibutilide, a New Compound with Potent Class Ill Antiarrhythmic Activity, Activates a Slow Inward Na+ Current in Guinea Pig Ventricular Cells’, 262(1), pp. 99–108.

Lee, K. S. and Lee, E. W. (1998) ‘Ionic mechanism of Ibutilide in human atrium: Evidence for a drug- induced Na+ current through a nifedipine inhibited inward channel’, *Journal of Pharmacology and Experimental Therapeutics*, 286(1), pp. 9–22.

Loewe, A. *et al.* (2014) ‘In-silico assessment of the dynamic effects of amiodarone and dronedarone on human atrial patho-electrophysiology’, *Europace*, 16, pp. iv30–iv38. doi: 10.1093/europace/euu230.

Loewe, A. *et al.* (2015) ‘Understanding the cellular mode of action of vernakalant using a computational model: Answers and new questions’, *Current Directions in Biomedical Engineering*, 1(1), pp. 418–422. doi: 10.1515/cdbme-2015-0101.

Maleckar, M. M. *et al.* (2009) ‘K+ current changes account for the rate dependence of the action potential in the human atrial myocyte’, *American Journal of Physiology - Heart and Circulatory Physiology*, 297(4), pp. H1398–H1410. doi: 10.1152/ajpheart.00411.2009.

Muszkiewicz, A. *et al.* (2018) ‘From ionic to cellular variability in human atrial myocytes: An integrative computational and experimental study’, *American Journal of Physiology - Heart and Circulatory Physiology*, 314(5), pp. H895–H916. doi: 10.1152/ajpheart.00477.2017.

O’Hara, G. *et al.* (1992) ‘Effects of flecainide on the rate dependence of atrial refractoriness, atrial repolarization and atrioventricular node conduction in anesthetized dogs’, *Journal of the American College of Cardiology*. Elsevier Masson SAS, 19(6), pp. 1335–1342. doi: 10.1016/0735-1097(92)90342-K.

Oshikawa, N. *et al.* (2001) ‘Frequency-dependent electrophysiological effect of ibutilide on human atrium and ventricle’, *Journal of Interventional Cardiac Electrophysiology*, 5(1), pp. 81–87. doi: 10.1023/A:1009866126492.

Quinteiro, R. A. and Biagetti, M. O. (1994) ‘Chronic Versus Acute Effects of Amiodarone on the Vmax-Conduction Velocity Relationship and on the Space Constant in Canine Myocardium’, *Journal of Cardiovascular Pharmacology*, 24(1), pp. 122–133.

Sánchez, C. *et al.* (2017) ‘Atrial fibrillation dynamics and ionic block effects in six Heterogeneous human 3D virtual atria with distinct repolarization dynamics’, *Frontiers in Bioengineering and Biotechnology*, 5(MAY), pp. 1–13. doi: 10.3389/fbioe.2017.00029.

Sun, W., Sarma, J. S. M. and Singh, B. N. (1999) ‘Electrophysiological Effects of Dronedarone (SR33589), a Noniodinated Benzofuran Derivative, in the Rabbit Heart Comparison With Amiodarone’, *Circulation*, 100(22), pp. 2276–2281.

Sun, W., Sarma, J. S. M. and Singh, B. N. (2002) ‘Chronic and acute effects of dronedarone on the action potential of rabbit atrial muscle preparations: Comparison with amiodarone’, *Journal of Cardiovascular Pharmacology*, 39(5), pp. 677–684. doi: 10.1097/00005344-200205000-00008.

Sutanto, H. *et al.* (2019) ‘Maastricht antiarrhythmic drug evaluator (MANTA): A computational tool for better understanding of antiarrhythmic drugs’, *Pharmacological Research*. Elsevier, 148(April), p. 104444. doi: 10.1016/j.phrs.2019.104444.

Suzuki, T. *et al.* (2013) ‘Atrial selectivity in Na channel blockade by acute amiodarone’, *Cardiovascular Research*, 98(1), pp. 136–144. doi: 10.1093/cvr/cvt007.

Wang, J. *et al.* (1993) ‘Comparative mechanisms of antiarrhythmic drug action in experimental atrial fibrillation: Importance of use-dependent effects on refractoriness’, *Circulation*, 88(3), pp. 1030–1044. doi: 10.1161/01.CIR.88.3.1030.

Wang, L. *et al.* (2007) ‘Cardiac glycosides as novel inhibitors of human Ether-a-gogo-related gene channel trafficking’, *Journal of Pharmacology and Experimental Therapeutics*, 320(2), pp. 525–534. doi: 10.1124/jpet.106.113043.

Wang, Z. *et al.* (1990) ‘Effects of flecainide and quinidine on human atrial action potentials’, *Circulation*, 82(1), pp. 274–283.

Wang, Z., Feng, J. and Nattel, S. (1995) ‘Idiopathic atrial fibrillation in dogs: Electrophysiologic determinants and mechanisms of antiarrhythmic action of flecainide’, *Journal of the American College of Cardiology*, 26(1), pp. 277–286. doi: 10.1016/0735-1097(95)90845-F.

Wettwer, E. *et al.* (2013) ‘The new antiarrhythmic drug vernakalant: Ex vivo study of human atrial tissue from sinus rhythm and chronic atrial fibrillation’, *Cardiovascular Research*, 98(1), pp. 145–154. doi: 10.1093/cvr/cvt006.

Wilhelms, M. *et al.* (2013) ‘Benchmarking electrophysiological models of human atrial myocytes’, *Frontiers in Physiology*, 3 JAN(January), pp. 1–16. doi: 10.3389/fphys.2012.00487.

Zeemering, S. *et al.* (2018) ‘The electrocardiogram as a predictor of successful pharmacological cardioversion and progression of atrial fibrillation’, *Europace*, 20(7), pp. e96–e104. doi: 10.1093/europace/eux234.
